# Role of Structural Dynamics at the Receptor G Protein Interface for Signal Transduction

**DOI:** 10.1371/journal.pone.0143399

**Published:** 2015-11-25

**Authors:** Alexander S. Rose, Ulrich Zachariae, Helmut Grubmüller, Klaus Peter Hofmann, Patrick Scheerer, Peter W. Hildebrand

**Affiliations:** 1 Institute of Medical Physics and Biophysics (CC2), Universitätsmedizin Berlin, Charitéplatz 1, 10098, Berlin, Germany; 2 Team ProteiInformatics, Universitätsmedizin Berlin, Charitéplatz 1, 10098, Berlin, Germany; 3 Team Protein X-ray Crystallography and Signal Transduction, Charité - Universitätsmedizin Berlin, Charitéplatz 1, 10098, Berlin, Germany; 4 Centre of Biophysics and Bioinformatics, Humboldt-Universität zu Berlin, Invalidenstrasse 42, 10115, Berlin, Germany; 5 Dep. of Theoretical and Computational Biophysics, Max-Planck-Institute for Biophysical Chemistry, 37077, Göttingen, Germany; 6 Computational Biology, School of Life Sciences, and Physics, School of Science and Engineering, University of Dundee, Dow Street, Dundee, DD1 5EH, United Kingdom; Russian Academy of Sciences, Institute for Biological Instrumentation, RUSSIAN FEDERATION

## Abstract

GPCRs catalyze GDP/GTP exchange in the α-subunit of heterotrimeric G proteins (Gαßγ) through displacement of the Gα C-terminal α5 helix, which directly connects the interface of the active receptor (R*) to the nucleotide binding pocket of G. Hydrogen–deuterium exchange mass spectrometry and kinetic analysis of R* catalysed G protein activation have suggested that displacement of α5 starts from an intermediate GDP bound complex (R*•G^GDP^). To elucidate the structural basis of receptor-catalysed displacement of α5, we modelled the structure of R*•G^GDP^. A flexible docking protocol yielded an intermediate R*•G^GDP^ complex, with a similar overall arrangement as in the X-ray structure of the nucleotide free complex (R*•G^empty^), however with the α5 C-terminus (GαCT) forming different polar contacts with R*. Starting molecular dynamics simulations of GαCT bound to R* in the intermediate position, we observe a screw-like motion, which restores the specific interactions of α5 with R* in R*•G^empty^. The observed rotation of α5 by 60° is in line with experimental data. Reformation of hydrogen bonds, water expulsion and formation of hydrophobic interactions are driving forces of the α5 displacement. We conclude that the identified interactions between R* and G protein define a structural framework in which the α5 displacement promotes direct transmission of the signal from R* to the GDP binding pocket.

## Introduction

**Fig 1:**
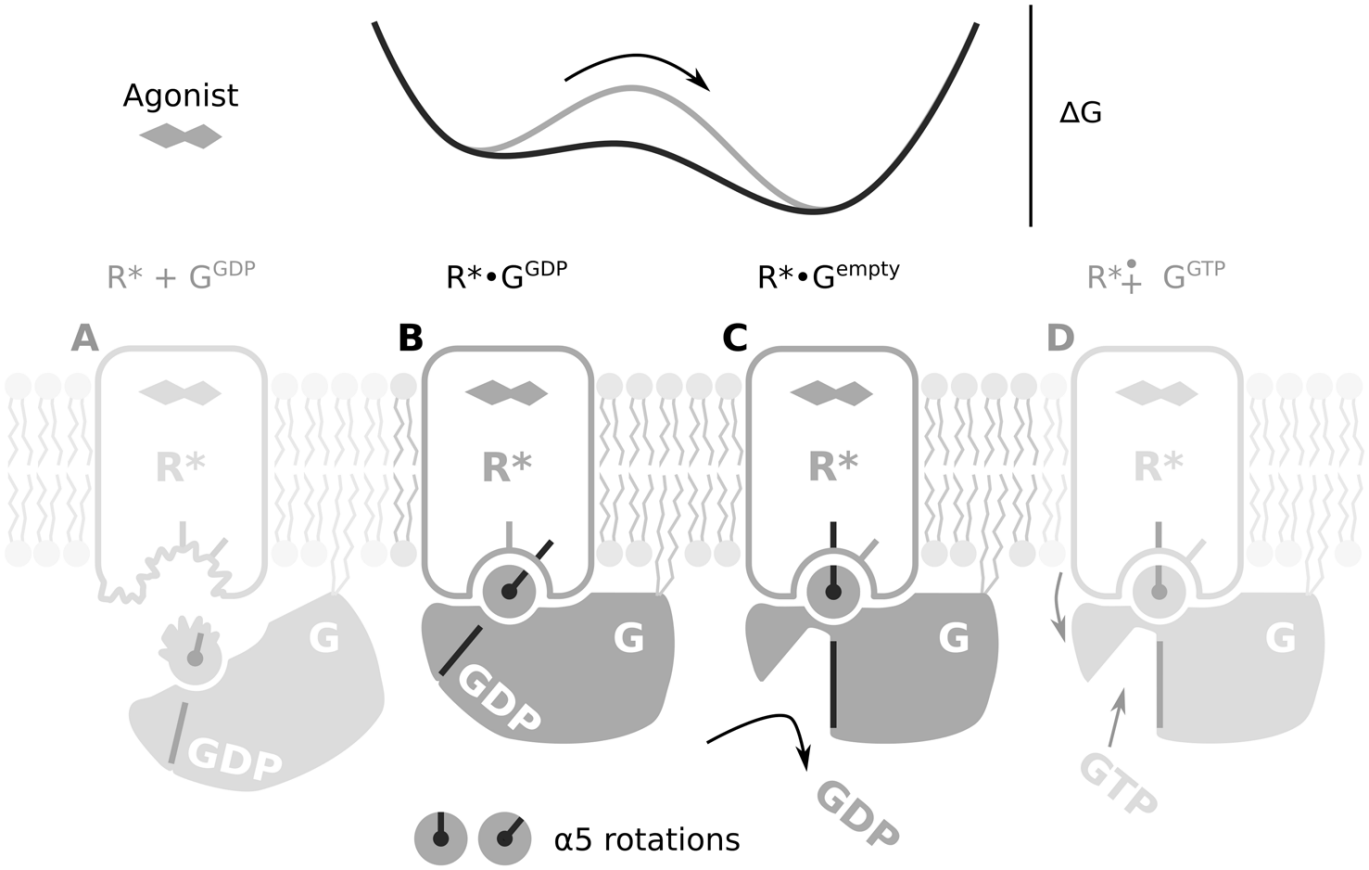
Role of the α5 helix in the interaction between R* and G that leads to nucleotide exchange. From left to right. (A) Membrane anchored G^GDP^ with an unstructured α5 C-terminus encounters R* with a partially unstructured cytoplasmic crevice. (B) The intermediate R*•G^GDP^ complex is formed through mutual structuring of the α5 C-terminus and the R* cytoplasmic crevice. The α5 helix has not yet rotated compared to unbound G^GDP^. (C) Rotation of α5 lowers the energy barrier separating R*•G^GDP^ from nucleotide free R*•G^empty^ resulting in GDP release. (D) Uptake of GTP and dissociation of G^GTP^ completes the nucleotide exchange reaction.

G protein coupled receptors (GPCRs) transmit extracellular signals into the cell through binding of heterotrimeric G proteins (Gαßγ, classified as Gi, Gt, Gs, …) and catalysing GDP/GTP exchange in the Gα subunit (Fig 1). Detailed insights into the structural changes triggering GDP release were obtained from recent X-ray structures of inactive (R) and active receptor states (R*) [1], of GDP bound G proteins (G^GDP^) and the nucleotide free R*•G complex (R*•G^empty^) [2,3]. A rearrangement within the 7-transmembrane helix (7-TM) bundle opens a cytoplasmic crevice in R* for binding of G^GDP^ [1,3–6]. In unbound G^GDP^, the nucleotide is tightly bound by the Gα helical and Ras domain enveloping the nucleotide [7]. In the R*•G^empty^ complex, the Ras domain α5 helix (α5) and the helical domain of Gα are displaced and the nucleotide pocket is emptied (Fig 1C). The α5 helix, which forms a direct linkage between the cytoplasmic R* crevice and the nucleotide free binding pocket, is considered the principal structural element in transmission of the signal from R* to the nucleotide binding pocket [8–10]. The mechanism by which R* triggers the displacement of α5 is thus key to understanding the catalytic function of GPCRs.

New insights into the structural basis for nucleotide exchange came from recent multi-microsecond MD simulations and DEER measurements [10]. These experiments show that displacement of the helical domain occurs spontaneously without GDP release in G^GDP^ even in the absence of R*. However, α5 is spontaneously only displaced when GDP has been omitted, or when the effect of R* is mimicked by restraining α5 in the R*•G^empty^ position. These observations corroborate the important role of α5 for signal transmission. The key question of the mechanism of α5 displacement by R*, and the course of events leading to GDP release, however, still remains to be elucidated.

Simulation of the complete process of receptor G protein association and catalysis of GDP/GTP exchange takes milliseconds and is thus presently still beyond computationally accessible time scales [10–12]. However, the characterisation of structural intermediates and simulations of intermediate steps are promising approaches to obtain insights into the structural mechanism of R* catalysed nucleotide exchange. Here, we focus on the R*•G^GDP^ intermediate complex (Fig 1B), which is visited during the progression from R* and G^GDP^ (Fig 1A) to the R*•G^empty^ complex (Fig 1C). Evidence for the R*•G^GDP^ complex, in which both the receptor and GDP are bound to the G protein, has been found in the kinetics of Gt activation in rod disc membranes [13,14] and in hydrogen—deuterium exchange mass spectrometry (HDX) data of Gs activation by the activated β_2_ adrenoceptor (β_2_AR*) [15]. Formation of R*•G^GDP^ pre-complexes seems to affect the specificity and kinetics of signal transmission [16,17] and may also play an important mechanistic role in R* catalysed signal transfer. In this study, we present a computational investigation of the structure and function of the R*•G^GDP^ complex, which has not been resolved by protein X-ray crystallography. We will focus on the question how this complex relates to the displacement of α5 upon G protein activation, which we have previously termed α5 helix-switch [14] (Fig 1B and 1C).

Upon binding to R^3.50^ within the conserved D[E]RY motif at the floor of the cytoplasmic crevice of R*, the far C-terminus of α5 (termed GαCT) adopts the structure of a continuous α-helix capped by a C-terminal reverse turn [3,5,18–22]. FTIR spectroscopy and MD simulations have elucidated the mutual structuring of the involved binding interfaces (indicated in Fig 1A–1C) [18,23–25]. Recent atomic-level simulations show that the structured conformation of GαCT can also occur in absence of R* [10]. In HDX experiments of Gs activation by the β_2_AR* (β_2_AR*•Gs), deuterium exchange rates increase only slightly at the Gs α5 C-terminus (GsαCT) after adding GDP to the nucleotide free preparation, and the low exchange rates indicate structured elements. In contrast, adding the non-hydrolyzable GTP analog GTPγS uncouples Gs from β2AR*•Gs^empty^ and results in high exchange rates of GsαCT [15]. These data suggest that in the β_2_AR*•Gs^GDP^ intermediate complex, GsαCT already adopts a helical conformation, stabilized by interactions with β2AR*. The HDX experiments have also indicated that, on the N-terminal end of α5, the contacts that stabilize GDP in its binding pocket are preserved in the β_2_AR*•Gs^GDP^ intermediate. Summarizing these measurements, it seems reasonable that in R*•G^GDP^ α5 is not yet displaced and that the overall topology of the Ras domain of G^GDP^ is preserved. Alignment of Gα^GDP^ with X-ray structures representing nucleotide free complexes, however, results in major clashes of Gα^GDP^ with R* and the membrane (see e.g. Fig 5 of ref. [5] or Fig S13 of ref. [10]). Thus, α5 must bind with a different orientation to R* in the GDP bound compared to the nucleotide free complex to produce a clash free arrangement.

The present investigation was motivated by our previous finding [14] that flexible docking of GtαCT 15- and 19-mer peptides to active rhodopsin (RhR*) yielded two different poses. The first pose recovered the topology of the RhR*•GtαCT X-ray structure (i.e. the most likely position of α5 in R*•Gi/t^empty^ [25,26]). Alignment of G^GDP^ to a second pose of GtαCT resulted in a clash free complex with RhR*, which we assigned to the intermediate RhR*•Gt^GDP^ complex [14]. In the present analysis of Gs^GDP^ binding to β_2_AR*, we applied the same docking protocol to GsαCT and β_2_AR*. We again detect one pose, which recovers the topology of β_2_AR*•Gs^empty^ and a second pose yielding β_2_AR*•G^GDP^. Both models of β_2_AR*•G^GDP^ and RhR*•G^GDP^ closely resemble the overall arrangement of the R*•G^empty^ complex, except that α5 forms distinct interactions with R*. The main change in the interactions of GDP bound and empty complexes triggers a rotation of α5 by about 60° within the cytoplasmic crevice of R*, which is exactly the value by which α5 is displaced during activation [9,26]. Our model suggests that the α5 displacement occurs within a fixed structural framework defined by the interactions between intracellular loop 2 (ICL2) of R* and structural elements of the G protein. To study the role of R* for the displacement of α5, we conducted MD simulations of GsαCT and GtαCT in the cytoplasmic crevices of β_2_AR* and RhR*, respectively, from our intermediate R*•G^GDP^ complexes. Reformation of hydrogen bonds, water expulsion and formation of hydrophobic interactions are found as the forces that drive the characteristic displacement of α5 within the cytoplasmic crevice of R*.

## Results

### A docking pose of a GsαCT peptide that belongs to the β_2_AR*•G^GDP^ complex

The first goal of this study was to model the GDP bound intermediate β_2_AR*•Gs^GDP^ complex that is visited in the progression from β_2_AR* and Gs^GDP^ to the R*•G^empty^ complex. Structural intermediates generally form towards the end of the protein association pathway after the rate-limiting step [27], which in case of the fast signal transfer from receptors to G proteins involve folding of GαCT and ICL3 [18,24]. We therefore applied flexible docking with fixed α-helical backbone geometry but flexible side chains of a 15-mer GsαCT peptide to the cytoplasmic crevice of β_2_AR* (Protocol C in S1 File). The coordinates of both the peptide and the receptor were extracted from the X-ray structure of the β_2_AR*•G^empty^ complex.

**Fig 2:**
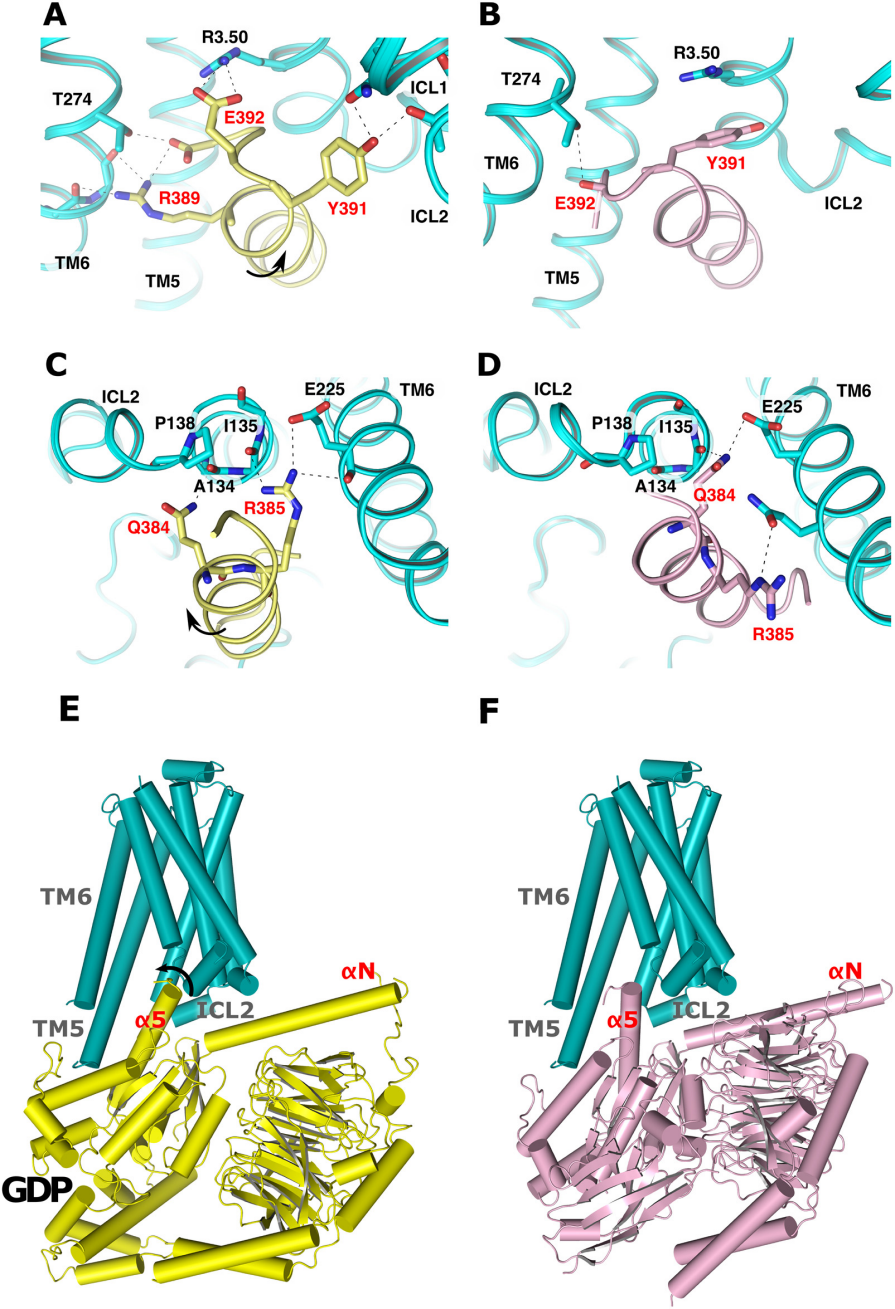
Comparison of the β_2_AR*•Gs^GDP^ model (left panel) and the β_2_AR*•Gs^empty^ X-ray structure (right panel). The figure illustrates potential hydrogen bonds to residues within the cytoplasmic crevice (cyan cartoon) from **(A, B)** the C-terminal reverse turn and **(C, D)** the N-terminus of GsαCT. **(A, C)** shows the intermediate position obtained from flexible docking of 15-mer GsαCT (yellow cartoon) and **(B, D)** the position in the nucleotide free complex (magenta cartoon), respectively. Residue labels from β_2_AR* are colored in black, from GsαCT in red. Potential hydrogen bonds are denoted as black dashed lines. **(E)** Complete model of the β_2_AR*•Gs^GDP^ intermediate compared to **(F)** the β_2_AR*•Gs^empty^ X-ray structure (PDB entry 3SN6). R*•G^GDP^ was obtained by superposition of Gsα^GTPγS^ (PDB entry 1AZT) with the intermediate β_2_AR*•GsαCT complex by common backbone atoms. Black arrows indicate the rotation of α5.

As in our previous analysis of GtαCT docking to RhR* [14], the highest scored docking pose of 15-mer GsαCT to β_2_AR* confirms the position and orientation seen in the X-ray structure of the nucleotide free complex (Fig G in S1 File; first cluster: 11 of 110 poses). In this pose, the characteristic cation-π interaction observed in β_2_AR*•Gs^empty^ is formed between Y391 of the C-terminal cap of GsαCT to R131^3.50^ of β_2_AR* (Fig 2B). Moreover, specific contacts are formed between the N-terminus of GsαCT with ICL2 and ICL3 of β_2_AR* (Fig 2D). These contacts involve potential hydrogen bonds of Q384 of GsαCT with the main chain carbonyl group of I135^3.54^ from the conserved P138^3.57^ helix cap motif (Table A of S1 File), which terminates TM3 of β_2_AR*. In a second docking pose (Fig G of S1 File; third cluster: 6 of 110 poses) the contact of GsαCT with R131^3.50^ is shifted C-terminally by one residue from Y391 to E392 (Fig 2A and 2B). N-terminally, the contact of GsαCT with the main chain carbonyl group of I135^3.54^ is also shifted by one residue from Q384 to R385 (Fig 2C and 2D; Table A of S1 File). We thus obtain not only the pose of GsαCT displayed by the X-ray structure (which validates the applicability of the docking approach) but also a second pose, in which GsαCT is rotated around its axis.

In the two poses, GsαCT binds to the same conserved structural motifs of β_2_AR*, however via different residues. Comparison of the two poses reveals that the shift of the interacting residues by one position entails a rotation of GsαCT by 60° and a translation by 1.5 Å (see Protocol E in S1 File). Of note, a similar rotation has also been observed when the two poses obtained from flexible docking of GtαCT to RhR* were compared [14]. Since Gs α5 undergoes a rotation of the same magnitude during G protein activation (Fig F in S1 File), superposition of nucleotide bound states of Gsα^GTPγS^ (PDB entry 1AZT) or Gt^GDP^ (PDB entry 1GOT) with GsαCT of that second pose creates a complex with a very similar overall arrangement as in β_2_AR*•Gs^empty^, but in which α5 is rotated by 60° (Fig 2E and 2F). As in our previous analysis of the RhR* interaction with Gt^GDP^, this new state does not cause any major protein-protein/protein-membrane clashes or distortions. Since α5 is also structured and Gs^GDP^ is not altered in that complex (see Fig 2E), it is assigned to the β_2_AR*•Gs^GDP^ complex. As a result of our docking analysis, we predict that the α5 helix-switch is triggered through sequential interactions of α5 with the cytoplasmic crevice of R*.

### Observation of helix-switches by molecular dynamics simulations

To evaluate the forces which guide the α5 helix-switch from the GDP bound to the nucleotide free state, we started MD simulations from the intermediate β_2_AR*•GsαCT and RhR*•GtαCT complexes. Using the R*•GαCT complexes ensures that only the interactions of GαCT with R*, but not of α5 with the remainder of the G holo protein play a role. Most simulations were performed with 11-mer GαCT peptides, as it is the largest common structure in the available experimental data [3,5,18]. To evaluate the effect of peptide length, we performed additional simulations with 19-mer GαCT peptides, in total 4 simulations for β_2_AR*•GsαCT and 6 for RhR*•GtαCT. During all MD simulations, GαCT was neither restricted to its starting conformation nor in its mobility.

**Fig 3:**
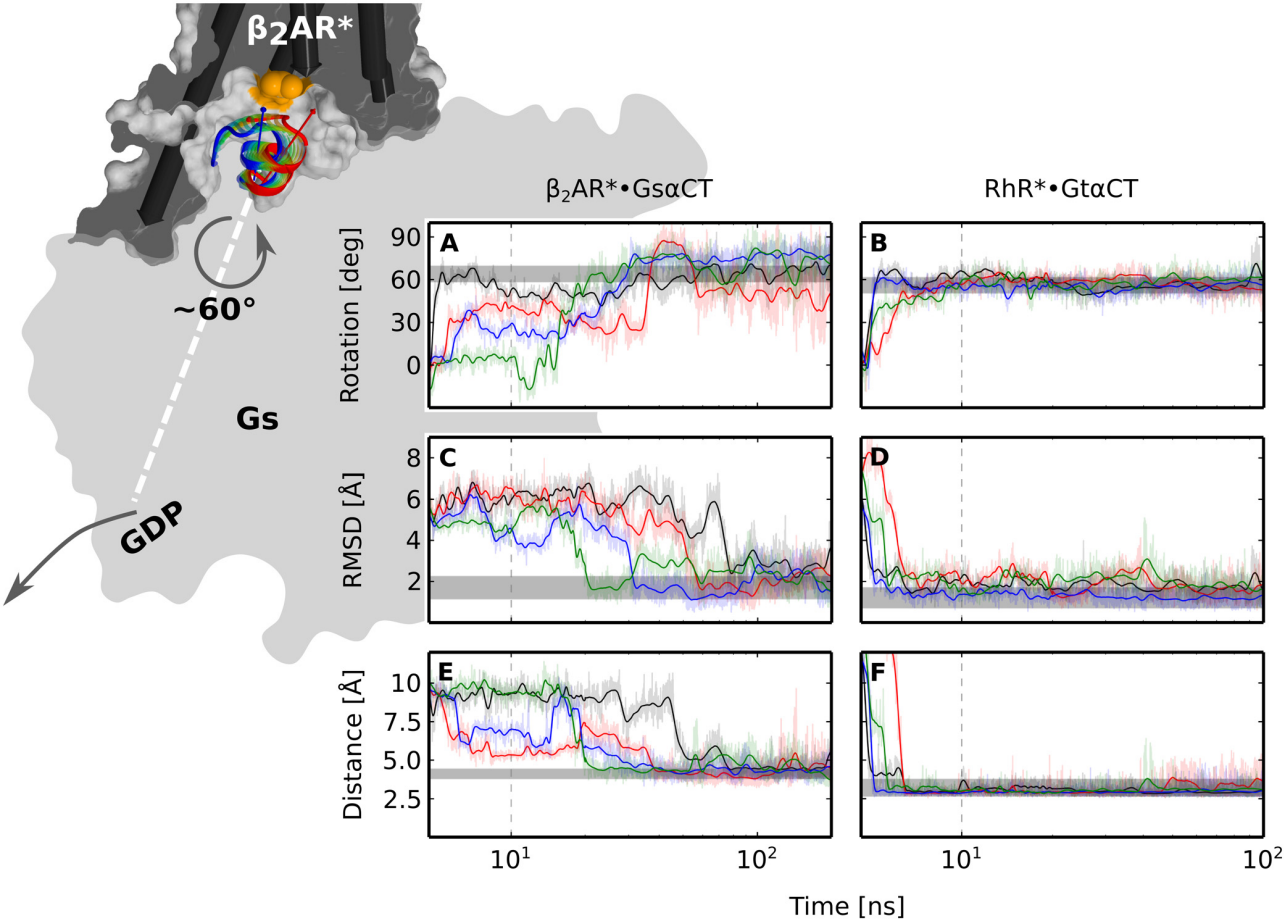
Switch of GsαCT (left) and GtαCT (right) at the R* interface observed in MD simulations. Background figure: GsαCT switches within the cytoplasmic crevice of β_2_AR* from the intermediate (red) to the nucleotide free position (blue). The transition is schematically indicated by semi-transparent colored cartoons. GsαCT is rotated around its helix axis (red and blue arrows) by about 60°, which eventually triggers GDP release from the nucleotide binding pocket of the Gs holoprotein (gray, flat shaded). In addition a tilt motion of GsαCT parallel to the membrane plane is observed. The surface of the receptor (gray) is cut at the position of R^3.50^ (orange patch) located at the floor of the cytoplasmic crevice. TM helices are shown as cylinders. For clarity, H8 and H6 of β_2_AR* are omitted. The panel in the foreground shows rotation of **(A)** GsαCT or **(B)** GtαCT around its helix axis; backbone-RMSD of **(C)** GsαCT or **(D)** GtαCT relative to the position in the X-ray structure; distance between **(E)** the center of the phenyl ring of Y391 of GsαCT and R131^3.50^ or **(F)** between the carbonyl oxygen of C347 of GtαCT and R135^3.50^. Gray bars indicate the range of mobility of GαCT in MD simulations of the X-ray structures of (left) holo β_2_AR*•Gs^empty^ (taken from ref. [25]) or (right) RhR*•GtαCT (see Figs B, C and E in S1 File; see Methods section). The mobility of switched GsαCT (after about 100 ns) is only slightly increased, when compared to the mobility of the corresponding section in β_2_AR*•Gs^empty^ (grey). The time series data are drawn on top of the raw data as a running average. The plots are linear for the first 10 ns and logarithmic for the remaining time (gray dashed lines). The four representative simulations (black, red, blue, green) of 11-mer GsαCT (Panel A of Fig N in S1 File, simulations 8, 9, 21 and 23) and of 11-mer GtαCT (Panel B of Fig N in S1 File, simulations 9, 16, 21 and 30) were picked from 8 and 10 simulations were a helix-switch was observed (Fig N in S1 File).

From the total of 60 MD simulations of 11-mer GαCT, 30*200 ns for the β2AR* and 30*100 ns for RhR* systems, 18 feature a helix-switch, 8 for GsαCT and 10 for GtαCT. In these simulations, the 11-mer adopts a conformation maintained for the remainder of the simulation (Figs N, O, P, Q and R in S1 File). These 'stable' binding modes form quickly, within 50 ns in case of GsαCT and within 3 ns in case of 11-mer GtαCT. In these simulations we observe a switch-like transition to the position seen in the corresponding X-ray structures. The different time scales observed for the transitions likely originate from the different properties of the corresponding binding interfaces. The larger interface of 11-mer GsαCT with the β_2_AR* (62.7 Å^2^) as of GtαCT with RhR* (35.9 Å^2^) in the intermediate, presumably slows down the switch motion. This relatively small difference of about 50 ns may not be relevant for the holo G protein, where additional contacts of α5 with G^GDP^ slow down the α5 helix-switch to the microsecond scale [10]. All transitions were monitored by the parameters peptide backbone-RMSD (Fig 3A and 3B and Fig N in S1 File) and peptide rotation (Fig 3C and 3D and Fig N in S1 File).

Despite the fact that two different receptors and peptides were simulated, the two GαCT peptides undergo similar screw-like motions to re-establish the key interactions with R^3.50^ seen in the respective X-ray structures. Formation of these latter interactions, which belong to the nucleotide free state, becomes apparent in the time resolved analyses of distance and energy (Fig 3E and 3F, Fig O in S1 File). Whilst the secondary structure of GαCT remains stable (Fig P in S1 File), a significant number of water molecules are displaced from the binding interface in favour of a hydrophobic patch that is formed between GαCT and ICL3 of R* (Figs K, Q and R in S1 File). The water molecules that remain bound to the interface in the MD simulation coincide with water molecules resolved in X-ray structures of RhR*•GtαCT (Fig S in S1 File). In the simulations in which no stable binding mode is observed, GαCT fluctuates between different orientations and positions, diffuses away and unfolds. Only one additional stable binding mode of GtαCT is obtained in simulations 11 and 12 (Panel B of Figs N, O, P, Q and R in S1 File). In these simulations, GtαCT is tilted steeper by 10° relative to the membrane plane compared to those in which a complete switch event is observed.

The simulations of 19-mer GαCT show a similar result to the 11-mer peptides (Panels B and C of Fig J in S1 File). Two helix-switch events were observed for each of the systems (RhR* and β_2_AR*) after about 50 ns of 200–400 ns simulation time, respectively, accounting for more than one third of the simulations. Presumably due to the lack of stabilizing contacts with R*, the first N-terminal turn of 19-mer GαCT undergoes helix-coil transitions during the switch (Panel A of Fig J in S1 File). By contrast, the secondary structure of the C-terminal reverse turn of switched GαCT is preserved and essentially immobilized through interactions with R* (grey bars in Fig 3A, 3C and 3E).

## Discussion

### Characterisation of the R*•G^GDP^ intermediate

Protein-protein interactions follow a multi-state process, from an initial encounter through an intermediate to the final functional complex [27]. Intermediates are thus envisioned as preformed complexes whose reorganization leads to the final functional complex. They form towards the end of the protein association pathway after the rate-limiting step, which in case of the fast signal transfer from receptors to G proteins involve a stepwise and mutual reduction of the conformational space of GtαCT and ICL3 [18,24]. Set after the rate limiting step, the C-terminal reverse turn and the ICL3 are completely structured in our models of R*•G^GDP^ intermediates. These intermediates already show a “double sandwich” structure comprising—in that order—the αN/β2-β3 loop of Gα, ICL2 of R*, α5 helix of Gα, and ICL3 of R* (Fig 2A, for GtαCT see [14]). This “sandwich” is also seen in the β2AR*•Gs^empty^ complex. In contrast to the β2AR*•Gs^empty^ complex, however, α5 is not displaced in the intermediates. We conclude that the interaction of the structural elements within the “double sandwich” structure generate a structural framework for the α5 helix-switch, which, according to our model, is guided by different sets of interactions with the cytoplasmic crevice of R*. However, we do not exclude that, in addition to α5, interactions of other elements of that structural framework, such as the αN/β2-β3 loop of Gα with ICL2 of R*, are involved in coupling of the R* interface with the nucleotide binding pocket.

The existence of the R*•G^GDP^ intermediate, in which GDP is still bound in the nucleotide binding site of the Gα subunit, was first derived from the kinetics of Gt activation in disc membrane [13,14] and HDX experiments of Gs binding to β_2_AR* [15]. In the latter work, Sunahara and co-workers provided first evidence for GDP bound Gsα which couples to β_2_AR* mainly through GsαCT. In the present analysis of the intermediate complex, GsαCT is bound to β_2_AR* through a strong hydrogen bond between E392 and R131^3.50^ of TM3 employing the E(D)RY motif. Additional hydrogen bonds are formed from GsαCT to ICL2 and 3 (Fig 2A–2C). Interactions with ICL2 are in line with previous work [28], where mutations of the ICL2 of rhodopsin lead to a receptor that binds but does not activate G protein, thus likely stabilizing an R*•G^GDP^ intermediate [29].

In the intermediate state models of both RhR* and β_2_AR*, neither GtαCT nor GsαCT exhibit extended hydrophobic interactions. The presence of transient polar interactions and the absence of stable hydrophobic interactions is indicative of intermediate states, as the full desolvation potential is not yet exploited [27]. In the context of receptor G protein coupling, it specifically reflects the weak binding of Gs^GDP^ to the receptor. In agreement with these characteristic properties, GsαCT in the intermediate position is highly dynamic in the MD simulations. The peptides either unfold and diffuse away or switch to the position of the nucleotide free complex within nanoseconds (Fig 2). Taking into consideration that the simulations were performed with the peptides and not with the holo G protein, it is likely that the switch of the α5 helix, which would be constrained by additional contacts with G^GDP^, occurs at considerably longer timescales. This assumption would be in line with the estimated microsecond lifetimes for intermediary states by the Frauenfelder model of protein dynamics [30].

### Intrinsic switching of the α5 helix at the R* interface

The two binding modes obtained from flexible docking analysis of GαCT to R* were assigned to the GDP bound and nucleotide free R*•G states. Comparison of these successive states indicates that the α5 helix-switching motion is promoted by consecutive interactions with the cytoplasmic crevice of R*. Further, MD simulations started from the putative GDP bound complexes show that this mechanism is structurally realizable, because the peptides very rapidly switch to adopt the stable position of the nucleotide free state. All key interactions present in the X-ray structures are eventually restored (Fig 3). This is not only seen for RhR*•GtαCT, for which an X-ray structure of R* with 11-mer GtαCT exists [5,31] but also for β_2_AR*•GsαCT, where additional contacts of Gs with β_2_AR* conceivably co-determine the position of α5 [3]. Thus, in principle no additional forces or constraints are required for GsαCT and GtαCT for transition from their intermediate states to the position and orientation they have in the nucleotide free states. Specifically, the only apparent forces are the interactions of the far C-terminus of α5 with the cytoplasmic crevice of R* and the expulsion of solvent. Thus, we argue that the ability of the α5 helix to switch is an intrinsic feature of the coupling interface comprising GαCT and the cytoplasmic crevice of R*. Following the theory of complex formation [27], transition from intermediates to the final functional states is guided by a reorganization of electrostatic interactions and dewetting of the interaction interfaces. In agreement with this notion, we observe new hydrogen bonds (Fig 2A–2D and Fig O in S1 File), expulsion of water, and formation of an extended hydrophobic contact patch at the binding interface of GαCT and ICL3 of R* (Figs K, Q and R in S1 File).

Formation of these new interactions presumably lowers the free energy required to reach the transition state, in which the contacts with the β2, β3, β5 and β6 half-barrel of G^GDP^ and the interaction with GDP, which lock α5 in its inactive position, are broken. At this specific point, our simulations complement recent microsecond MD simulations, where receptor-mimicking restraints led to displacement of α5 in GDP bound Gα [10]. While harmonic position restraints were applied in the former analysis to guide interface atoms of Gα (and thereby α5) to the positions of corresponding atoms in the β2AR*•Gs^empty^ crystal structure, our unbiased MD simulations explicitly reveal the reorganisation of contacts with R* that promote displacement of α5. Unlocking α5 from its inactive position results in a progressive downhill reaction, which includes displacement of the helical domain, disordering of the β1-α5 loop, breakage of the interactions stabilizing GDP and formation of new contacts between α5 and the Gα half barrel. Taken together, we conclude that the interactions of α5 with the R* interface are the key driving forces for its switching motion, which ultimately leads to GDP release.

### The α5 helix-switch involves highly conserved structural elements

In the mechanism proposed here, the α5 helix acts as a lever arm that transmits the signal from R* to the GDP binding pocket. The α5 helix thereby inherently exposes two adjacent sites to highly conserved motifs at R^3.50^ and at the P^3.57^ cap motif of TM3 (Fig 2, Fig I in S1 File and Table A in S1 File). Thus, two different (but not mutually distinct) interaction networks are sequentially engaged during the transition from R*•G^GDP^ to the R*•G^empty^ complex. Conceivably, these interaction networks could have a role for receptor G protein coupling specificity, when R* / Gα C-terminus complementarity is verified twice. The interaction network for R*•G^GDP^ would not require altering the position of α5 and would consequently be uncoupled from GDP release. Such a scenario is in line with the formation of a non-productive pre-coupled complex and with a role of the Gα C-terminus in controlling the kinetics and specificity of GPCR signalling pathways [16,17]. Indeed, the observed interaction between R131^3.50^ and the carboxyl group of E392 in the β_2_AR*•G^GDP^ complex would explain the key role of E392 in selective activation of Gs [32], because no contacts with its acidic side chain are observed in the X-ray structure of the β_2_AR*•Gs^empty^ complex. More generally, the involvement of the highly conserved motifs at R^3.50^ and at the P^3.57^ cap motif of TM3 (Fig S in S1 File; Panel A of Figs P and Q in S1 File; Table A in S1 File) in the hydrogen bond network between GαCT and R* indicates that the observed mechanism may apply in similar form to other GPCR/G protein systems.

## Conclusion

As the first specific complex during receptor-catalyzed nucleotide exchange, the R*•G^GDP^ intermediate pre-complex provides a structural framework, in which the α5 helix can act as a rod to transmit the signal from the activated receptor R* to the GDP binding pocket in the G protein α-subunit. Our simulations reveal the dynamic interactions which occur during the “helix-switch”. It is found that, starting from R*•G^GDP^, the C-terminal end of α5 undergoes a characteristic screw-like motion and reconstitutes all specific contacts of the nucleotide free R*•G^empty^ complex spontaneously and without external interaction. We conclude that the interactions of α5 with R* in the R*•G^GDP^ complex initiate a progressive downhill reaction which ultimately leads to GDP release.

## Methods

### Structure preparation

The structural models underlying the docking experiments and the MD simulations were prepared based on X-ray structures from co-crystals of β_2_AR* with Gsαβγ [3] and of RhR* with GtαCT [5]. The GtαCT binding cavities in the crystal structures of Ops*/ Meta II with (PDB entry 3DQB/ 3PQR) and without (PDB entry 3CAP/ 3PXO) GtαCT do not differ significantly from each other in backbone RMSD. We selected 3DQB as a representative of the active Meta II state of rhodopsin (termed RhR*). For all simulations involving β_2_AR*, the coordinates from the β_2_AR*•Gs^empty^ complex (PDB entry 3SN6) with the agonist bound but with the T4-lysozyme removed from the N-terminus, were used. To prepare the complex for simulation, unresolved side chain atoms were added and three mutated residues (M96T, M98T and N187E) in β2AR* were changed back to the wild-type form. The coordinates for the missing residues of ECL 2 (176–178) were taken from the nanobody-stabilized active β2AR*-structure (PDB entry 3P0G). The conformation of the residues 240 to 264 from ICL 3, which are not critical to receptor function [33], were modelled with help of the loop modeling program SuperLooper [34]. Of note, none of the modelled sections were part of the cytoplasmic crevice. Internal water molecules were added as described in the Protocol B of S1 File as well as the choice of appropriate protonation states.

### Flexible docking analysis

We applied the flexible docking protocol from our previous analysis, with fixed main chain but flexible side chain topologies of R* and GαCT. This allowed fast calculation of a large conformational space of possible GsαCT binding modes and conformations while staying close to experimentally determined structures. Flexible docking analysis of β_2_AR* and 15-mer GsαCT was performed with the program GOLD as described previously [14]. The docking program GOLD [35] is based on a genetic algorithm to explore a defined range of ligand conformational flexibility with partial flexibility of the receptor. The docking results of β_2_AR* and 15-mer GsαCT from 11 independent runs were clustered applying the single linkage method with a cut-off of 1.5 Å as implemented in the tool g_cluster of the program GROMACS (see Protocol C in S1 File for more information).

### Molecular dynamics simulations

We performed unbiased all-atom MD simulations of β_2_AR*/ RhR* and 11/19-mer GαCT with explicit water molecules and a lipid bilayer. The peptide length was derived from analysis of simulations of β_2_AR*•Gs / GsαCT complexes, which revealed that the binding interface with α5 maximally consists of 15 C-terminal residues [25]. Simulations were carried out with 11-mer peptides, reassembling the length of GtαCT used in binding assays and for X-ray crystallography and with 19-mer peptides, where the helicity of the 15-mer is preserved through hydrogen bonding with the N-terminal extension.

System preparation and subsequent minimization and equilibration were performed with the GROMACS suite (version 4.5) [36]. The prepared proteins (see Protocol A of S1 File) were inserted into the equilibrated bilayer of dimyristoylphosphatidylcholine (DMPC) using the GROMACS g_membed tool [37]. Parameters for the DMPC lipids were derived from Berger et al. [38] and for water from the SPC/E model [39]. A salt concentration of 0.15 mol/L was obtained by adding Na^+^ and Cl^−^ ions to the system with the GROMACS tool genion. The AMBER99SB-ILDN force field [40] was used for proteins and ions. Ligand parameters for the agonist *5-hydroxy-4H-benzo[1,4]oxazin-3-one* of β_2_AR* were created with the PRODRG2 webserver [41].

The simulation protocol consisting of energy minimization, equilibration and production runs was performed as described in Protocol D of S1 File. The MD simulations starting with GαCT from the position and orientation of the co-crystals consist of ten 200 ns MD runs for RhR*•GtαCT and β_2_AR*•GsαCT, respectively [25]. The 30 simulations starting from the respective complexes in the putative R*•G^GDP^ intermediate were 100 ns long for the RhR*/ GtαCT system and 200 ns for the β_2_AR*/ GsαCT system.

## Supporting Information

S1 File Includes Protocols A-G, Table A and Figures A-S. (PDF)
