# Supplementary material for: Role of Structural Dynamics at the Receptor G Protein Interface for Signal Transduction
Source: PLoS One. 2015 Nov 25;10(11):e0143399. doi: 10.1371/journal.pone.0143399 (PMC4659624; doi:10.1371/journal.pone.0143399)
Supplement: S1 File — (PDF) [file pone.0143399.s001.pdf]

S1 File

Protocol

Preparations of R* and GαCT structures and complexes

The structural models underlying the docking experiments and the MD simulations were prepared based on X-ray structures from co-crystals of β_2AR* with Gsαβγ [1] and of Ops* with GtαCT [2]. The GtαCT binding cavities in the crystal structures of Ops*/ Meta II with (PDB entry 3DQB/ 3PQR) and without (PDB entry 3CAP/ 3PXO) GtαCT do not differ significantly from each other in terms backbone RMSD. We selected 3DQB as a representative of the active Meta II state of rhodopsin (termed RhR*). For all MD simulations based on 3DQB two palmitoyl chains were attached to the residues C322 and C323. Two 200 ns control MD simulations of 3DQB with GtαCT removed show a backbone RMSD of the cytoplasmic crevice of 1.4 Å±0.42 (± denotes the standard deviation). The backbone RMSD of the binding pocket with 3CAP as the structural reference is 1.6 Å±0.49. This corroborates the similarity of the binding pocket in the crystal structures of Ops* with and without GtαCT. The C-termini of both R* structures lack a larger region (RhR*: residues 327 to 348, UniProt entry P02699; β_2AR*: 342-413, UniProt entry P07550) which is not resolved in any other structure but inactive rhodopsin (PDB entry 1U19). Because removal of this region has been reported not to affect activation of Gt [3] we did not model this part of R*.

For all simulations involving β_2AR*, the coordinates from the β_2AR*•Gsαβγ[empty] complex (PDB entry 3SN6) with the agonist bound but with the T4-lysozyme removed from the N-terminus, were used. A palmitoyl chain was ligated to C341 of R*. Unresolved atoms from the side chains of residues 63, 97-99, 101, 149, 175, 192-195, 267, 269-272, 299, 301-302, 304, 306 and 333 were added using standard geometries from the Dunbrack 2002 library [4]. Three mutated residues (M96T, M98T and N187E) in β_2AR* were changed back to the wild-type form. The coordinates for the missing residues of ECL 2 (176-178) were taken from the β_2AR*-structure (PDB entry 3P0G) in which ECL 2 is resolved [5]. The conformation of the residues 240 to 264 from ICL 3, which are not critical to receptor function [6], were modeled with help of the loop modeling program SuperLooper [7], which is based on a database of existing loops from the PDB (www.rcsb.org) linking transmembrane helices (Fig H).

For flexible docking [8] and simulations of GtαCT (G340-F350) we used the coordinates from the high-affinity K341L variant (PDB entry 3DQB), which binds in the same position and orientation as the double high-affinity peptide GtαCT2 (K341L and C347V, PDB entry 3PQR), to generate the structure of RhR* with native GtαCT (RhR*•GtαCT). The 15/19-mer GtαCT were created by extending 11-mer GtαCT N-terminally by an ideal α-helix. For

simulations of 11/19-mer GsαCT (residues 376/384-394) and flexible docking of 15-mer GsαCT (residues 380-394) the coordinates from the $β_2$AR*•Gsαβγ complex were used.

Modeling protonation states and internal water

The C-termini of GtαCT, GsαCT, RhR* and $β_2$AR* were deprotonated (COO^-), whereas the N-termini were fully protonated ($NH3^+$). In RhR*, D83 [9,10], E113 [11], E122 [10] and E134 [12] were protonated. In $β_2$AR*, E122 was protonated, because it is in close contact with the hydrophobic lipid tails at the middle of the bilayer (as suggested by Dror et al. [13]). All other amino acids were given their default protonation states.

To fill small internal cavities, buried water molecules in low energy states were added by means of the program DOWSER [14]. The retinal binding pocket of Ops* (i.e. used as a representative of RhR*) was filled with bulk waters. For that purpose, Ops* was completely embedded in water, and the amino acid side chains and water molecules were allowed to equilibrate during a 60 ns simulation, while the protein backbone atoms were restraint to their initial positions. As a result, water entered through the two openings that allow retinal exchange within the lipid bilayer under native conditions [15]. The steady state was reached after about 20 ns, with approximately 27 water molecules in the (ligand-free) retinal binding pocket.

Flexible docking analysis

To sample a larger space of possible GsαCT binding sites and conformations within the $β_2$AR* cavity, we applied the flexible docking protocol from our previous analysis [16]. The docking program GOLD [8] is based on a genetic algorithm to explore a defined range of ligand conformational flexibility with partial flexibility of the receptor. Docking of GtαCT with fixed α-helical backbone geometry to the cytoplasmic crevice of RhR* recovered the orientation observed in the X-ray structure of RhR*•GtαCT. Docking with longer 15- and 19-mer GtαCT revealed a second type of interaction in addition to the X-ray state, that was related to the position of α5 in an intermediate R*•G[GDP] complex, because G[GDP] can be superimposed with GtαCT without any major clashes of proteins or distortions of the membrane. Here we find an analogous pair of states by flexible docking of 15-mer GsαCT to $β_2$AR* (Figs 2 and G). As in our previous analysis, the backbone geometry of GsαCT and $β_2$AR* was fixed, but side chains from the peptide and the cytoplasmic crevice (T68, I135, Y219, E225, R228, Q229, T274, I278, Y326 and R328, see also Fig Ia) of $β_2$AR* were allowed to rotate freely during the docking process.

The 10 best ranked results from 11 independent docking runs were clustered applying the single linkage method with a cut-off of 1.5 Å as implemented in the tool g_cluster of the program GROMACS. Thereby 110 docking poses were clustered into 58 clusters. The largest cluster (11 poses) coincides with the position and orientation of GsαCT in the X-ray structure of $β_2$AR*•Gs[empty] (Fig G), but in a slightly shifted orientation which is in agreement with the shift obtained from MD simulations (Fig B panel A). In the largest cluster, GsαCT shows the characteristic cation-π interaction between Y391 and R131$^{3.50}$ (Fig 2). As in the X-ray structures and in most simulations of 11-mer GsαCT (see Fig D

panel A), contacts with ICL2 and 3 of β_2AR* are formed by the N-terminus of 15-mer GsαCT. This involves hydrogen bonds of Q384 with the main chain carbonyl group of I135 (Fig D), which is part of the C-terminal P138$^{3.57}$ cap that terminates TM3 and stabilizes ICL2 [17]. Our sequence analysis of class A GPCRs shows that P138$^{3.57}$ is with 74% conserved (Table A).

The orientation from the second largest cluster (9 poses) is incompatible with an R*•G[GDP] intermediate (Fig G), but the third largest cluster (6 poses) features a rotation analogous to the one found in our previous analysis [16] with RhR* and GtαCT. The C-terminal reverse turn in the third largest cluster is detached from TM6 and closer to ICL1 compared to the X-ray state (Figs 2 and G). Thereby GsαCT is stabilized by an interaction of Y391 with residues from a conserved polar cleft between ICL1/TM2 and H8 (Table A). In the third largest cluster, the reverse turn of GsαCT is shifted by one residue relative to R131$^{3.50}$, compared to the X-ray state. In lieu of the cation-π interaction with Y391, R131$^{3.50}$ can form a hydrogen bond with E392 (Fig B panels A, B). N-terminally, instead of a hydrogen bond to Q384, the backbone oxygen of I135 from the P138$^{3.57}$ cap can form a hydrogen bond to R385 (Fig B panels C, D). In the two states, GsαCT thus binds to both conserved structural motifs of β2AR*, but with its contacts shifted by one residue. As a result, GsαCT in the X-ray state differs from the third largest cluster by a rotation of about 60° (see *Calculation of helix axis, helix tilt and rotation in S1 File*). Since α5 undergoes a rotation of the same magnitude during GDP release (Fig F), superposition of nucleotide bound states of Gs or Gi/t with β2AR*•GsαCT in the orientation of the third largest cluster does not cause any major clashes of proteins or distortions of the membrane (Fig 2E) and we thus relate it to an intermediate R*•G[GDP] complex.

Molecular dynamics protocol

System preparation and subsequent minimization and equilibration were performed with the GROMACS suite (version 4.5) [18]. The proteins were inserted into the equilibrated bilayer of dimyristoylphosphatidylcholine (DMPC) using the GROMACS g_membed tool [19]. Parameters for the DMPC lipids were derived from Berger et al. [20] and for water from the SPC/E model [21]. A salt concentration of 0.15 mol/L was obtained by adding Na^+ and Cl^- ions to the system with the GROMACS tool genion. The AMBER99SB-ILDN force field [22] was used for proteins and ions. Ligand parameters for the agonist *5-hydroxy-4H-benzo[1,4]oxazin-3-one* of β_2AR* were created with the PRODRG2 webserver [23]. A second set of parameters was created using the acpype/GAFF method [24–26] and a 200 ns control simulation of β_2AR* showed no (considerable) changes in cytoplasmic crevice behavior.

The simulation protocol consists of an energy minimization of the system, followed by an equilibration step. Based on the equilibrated systems, series of production runs were started with different initial velocities obtained from a 320° K Boltzmann distribution. For equilibration and the production runs all bonds were constraint using the LINCS algorithm [27], with the exception of water bonds, which were constraint by the SETTLE algorithm [28]. The temperature was kept constant by coupling the system to a temperature bath of

320° K, which is high enough to keep the DMPC membrane from entering the gel phase. The temperature coupling was performed using the velocity-rescaling thermostat of Bussi et al. [29] with a time constant of 0.2. Long range electrostatics were calculated with the PME method [30]. Berendsen pressure coupling was performed with a time constant of 2.0 and semi-isotropic scaling separating scaling in the membrane plane directions from the z-direction (i.e the membrane plane normal). The integration time step used for all simulations was 0.002 ps.

Energy minimization of all systems was done in GROMACS using the steepest descent algorithm until the maximum force went below 1000.0 kJ/mol/nm. This provides clash-free structures suitable for MD simulations. In the equilibration step the energy minimized structure was simulated for 20 ns with position restraints on all protein backbone atoms to keep the atoms at their initial position. This step allows for relaxation at the protein-membrane, protein-water and the membrane-water interfaces so that any voids at these interfaces are filled and side chains at these interfaces adopt fitting conformations. The production MD simulations were run with the same parameters as the equilibration simulations but without the position restraints. The MD simulations starting with GαCT from the position and orientation of the co-crystals consist of ten 200 ns MD runs for RhR*•GtαCT and β_2AR*•GsαCT, respectively. The 30 simulations starting from the respective complexes in the putative R*•G[GDP] intermediate were 100 ns long for the RhR*/ GtαCT system and 200 ns for the β_2AR*/ GsαCT system.

Calculation of hydrogen bond and cation-π interaction energies

Potential hydrogen bonds between R135$^{3.50}$ of RhR* and C347.O of GαCT were monitored as a function of the distance between the acceptor atom C347.O and the hydrogen atoms: HE, HH11, HH12, HH21 and HH22 of R135$^{3.50}$ (as named in the AMBER99SB-ILDN force field). The hydrogen bond energies were calculated according to the formula of Espinosa as a function of the distance between the acceptor atom and the hydrogen of the donor atom: $-0.5\cdot(50\cdot 1.1\cdot 10^{3}\cdot e^{-36\cdot d})$, where d is the distance between acceptor and hydrogen [31]. The hydrogen bond interaction energy was calculated as the sum of the hydrogen bonding energies of all acceptor-donor pairs. The hydrogen bonds including the atoms HE and HH21 of R135$^{3.50}$ contribute most to the total energy.

Cation-π interactions between R131$^{3.50}$ of β_2AR* and Y391 of GsαCT were detected with the program CaPTURE [32]. This program also reports an electrostatic interaction energy estimate that agrees qualitatively with *ab initio* calculations at the HF/6-31G** level, but underestimates the magnitude by roughly a factor of two which is about the same as the van der Waals interaction energy that is also reported by this program [32]. Here we use the electrostatic interaction energy as reported by CaPTURE to qualitatively capture the trend of the cation-π interaction in the MD simulations.

Calculation of helix axis, helix tilt and rotation

The first turn of both GαCT peptides is highly dynamic, because of the absence of N-terminal intra-helical hydrogen bonding partners (stabilizing this part of the α-helix in α5)

(Fig A). The overall stability of the α-helical conformation of residues 3-8 and of the turn structure of residues 9-11, were assessed by secondary structure content (Fig A) using the program DSSP [33], which allows to determine representative helix axes for the two GαCT peptides and thus variations in tilt and rotation (Fig E).

The rotation and tilt movements of GtαCT and GsαCT were calculated from the local axis of their α-helical sections as implemented in the GROMACS tool g_helixorient [18]. The program calculates the local rotation and tilt for groups of four consecutive Cα atoms. We calculated these values for all α-helical sections and subsumed the local tilt and rotation respectively to get an overall measure of the rotation and tilt movement. The tilt angle between GαCT and the membrane plane is subsumed analogously as the angle between the local helix axis and its projection onto the membrane plane.

The membrane plane was defined from the position of R* relative to the initial lipid bilayer into which R* was inserted and was thus not dynamically recalculated during the MD simulations. Sample calculations of the membrane planes as the best fitting (least square) planes through all lipid head group phosphates confirmed that the orientation of R* within the lipid bilayer does not change much.

Calculation of GαCT RMSD and lateral motion within the cytoplasmic crevice of R*

Two different types of backbone root mean square deviation (RMSD) were calculated with the GROMACS tool g_rms. The cytoplasmic crevice RMSD describes its structural integrity and was calculated as the RMSD of the backbone atoms after a least square superposition to the backbone atoms of cytoplasmic crevice from the equilibrated system. The cytoplasmic crevice was defined as all residues of R* that are in contact (atoms within 4 Å) with residues from the C-terminal reverse turn of GsαCT (residues 391-393) and GtαCT (348-350). The peptide RMSD describes the movement of GαCT relative to its position in the crystal structure. It was calculated as the RMSD of the backbone atoms from GαCT after the same superposition as used to determine the RMSD cytoplasmic crevice. Because the N-terminal turn of GsαCT is very flexible (for DSSP analysis see Fig A) the first two residues at the N-terminus were omitted for RMSD calculations. The lateral (XY) motion of the C-terminal reverse turn within the membrane plane was calculated from the geometric center of the backbone atoms of the last seven C-terminal residues of GαCT.

β_2AR	Conservation	Location
T66	T 37%, S 14%, N 5%, K 4%, R 4%, A 4%, Q 3%, H 2%	ICL1
N69	Y 33%, N 29%, D 6%, T 4%, S 3%, A 2%, F 2%, H 2%, R 2%	ICL1/TM2
$R131^{3.50}$	R 89%, C 2%	TM3
$P138^{3.57}$	P 74%, A 10%, S 3%	TM3
D331	E 20%, D 19%, K 8%, Q 7%, N 7%, R 5%, T 3%, S 3%, G 2%, H 2%	H8

Table A. Conservation of residues from the cytoplasmic crevice of R* important for stabilization of the R*•G[GDP] intermediate. Conservation was calculated from the alignment of all sequences from the UniProt data base (http://www.uniprot.org/, release 2015_09) which belong to the g protein coupled receptor 1 family, are reviewed and have a maximum sequence identity of 90%. Sequence alignment was performed with the Clustal Omega program. Only amino acids observed in at least 2% of the aligned sequences are referred.

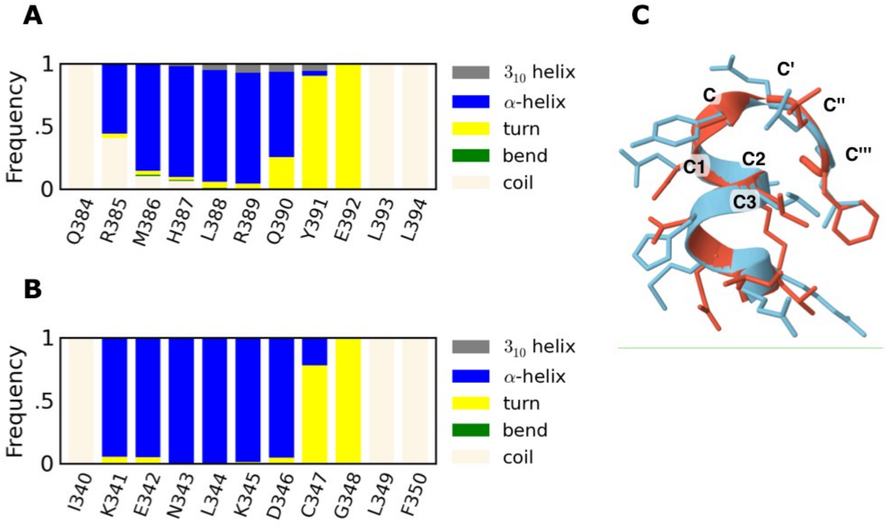

Fig A. Secondary structure analysis of GαCT in (A) β_2AR*•GsαCT and (B) RHR*•GtαCT. Starting from the conformation of the X-ray structures from the respective co-crystals, analysis with the program DSSP of ten 200 ns simulations shows stable helical core regions (blue), but helix-coil transitions at the N-terminus (pale orange). The first two residues were accordingly ignored for calculation of helix axes (Fig E) and backbone-RMSD (Fig B in S1). The C-terminus is defined by two residues forming a stable turn (yellow). Y391 or C347 (interacting with $R^{3.50}$ of the cytoplasmic crevice) are both in position C, the so defined last residue of the helix [34]. It proceeds E392 or G348 in position C' defined as the first turn residue. The last two C-terminal residues are assigned as coil by DSSP, but are part of the structured C-terminal reverse turn and thus were included in the analysis of backbone-RMSD. (C) Superposition of GsαCT and GtαCT. Labeling of the C-terminal reverse turn according to Aurora & Rose [34].

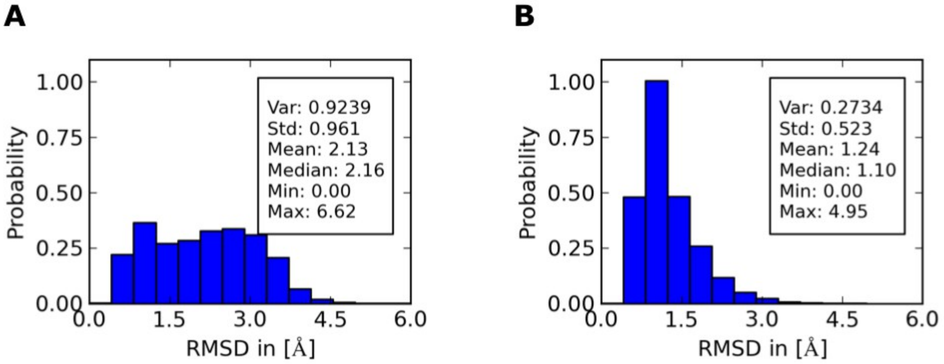

Fig B. Mobility of (A) β_2AR*•GsαCT and (B) RhR*•GtαCT monitored by the backbone-RMSD of GαCT. The GαCT backbone-RMSD describes the movement of GαCT relative to its position in the X-ray structures. It is calculated over the complete trajectory of the simulation, after superposition to the cytoplasmic crevice from the equilibrated system (also see *Calculation of cytoplasmic crevice and peptide RMSD and lateral peptide motion* in *S1 Protocol*). The histograms are calculated from ten 200 ns simulations each. For analysis only the last 9 residues were considered (for the explanation see legend Fig A).

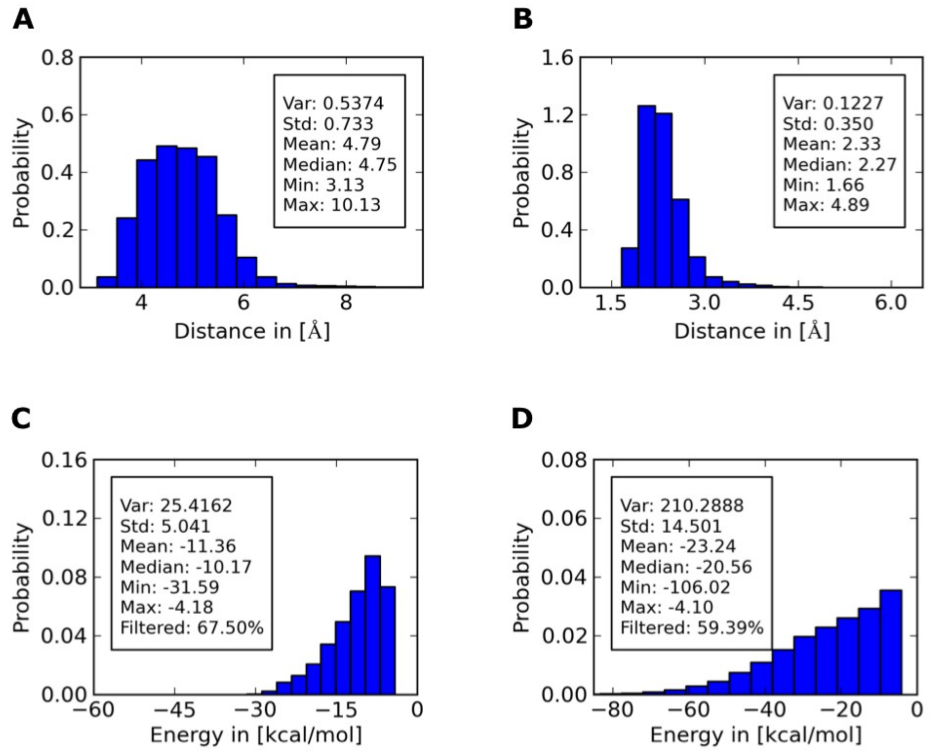

Fig C. Distance and interaction energies with R$^{3.50}$ in β$_2$AR*•GsαCT and RhR*•GtαCT. Distance between **(A)** the center of the phenyl ring of Y391 of GsαCT and R$^{3.50}$ of β$_2$AR* or **(B)** between the carbonyl oxygen of C347 of GtαCT and R$^{3.50}$ of RhR*. **(C)** Cation-π interaction energy between Y391 of GsαCT and R$^{3.50}$ of β$_2$AR* or **(D)** hydrogen bond energy between carbonyl oxygen of C348 of GtαCT and R$^{3.50}$ of RhR*. For clarity, only energies < −4.1 kJ/mol are shown. The histograms average over the ten 200 ns simulations, fully depicted in Fig M.

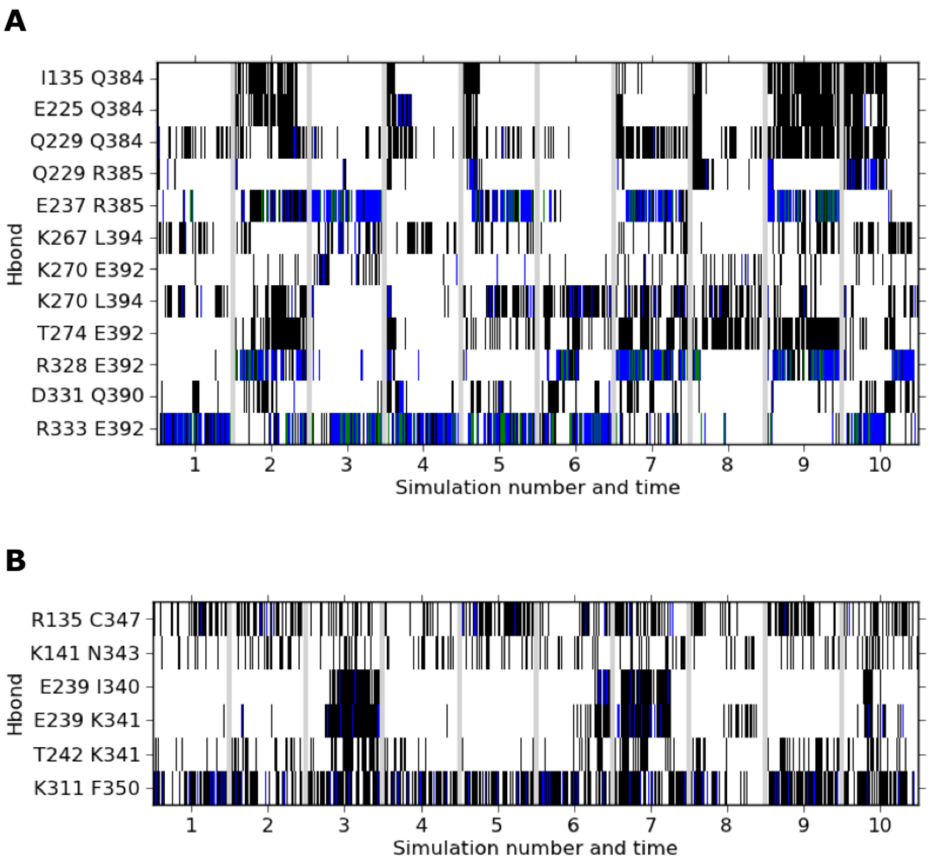

Fig D. Potential hydrogen bonds stabilizing (A) β_2AR*•GsαCT and (B) RhR*•GtαCT. Only those interactions with a donor-acceptor distance < 3.5 Å and an angle between acceptor-donor-hydrogen < 30° that persist for at least 10% of the trajectory of a simulation were considered as potential hydrogen bonds. The number of potential hydrogen bonds between two residues at a given point in time is color coded: black indicates one, blue two and green three hydrogen bonds. Grey bars separate independent 200 ns simulations.

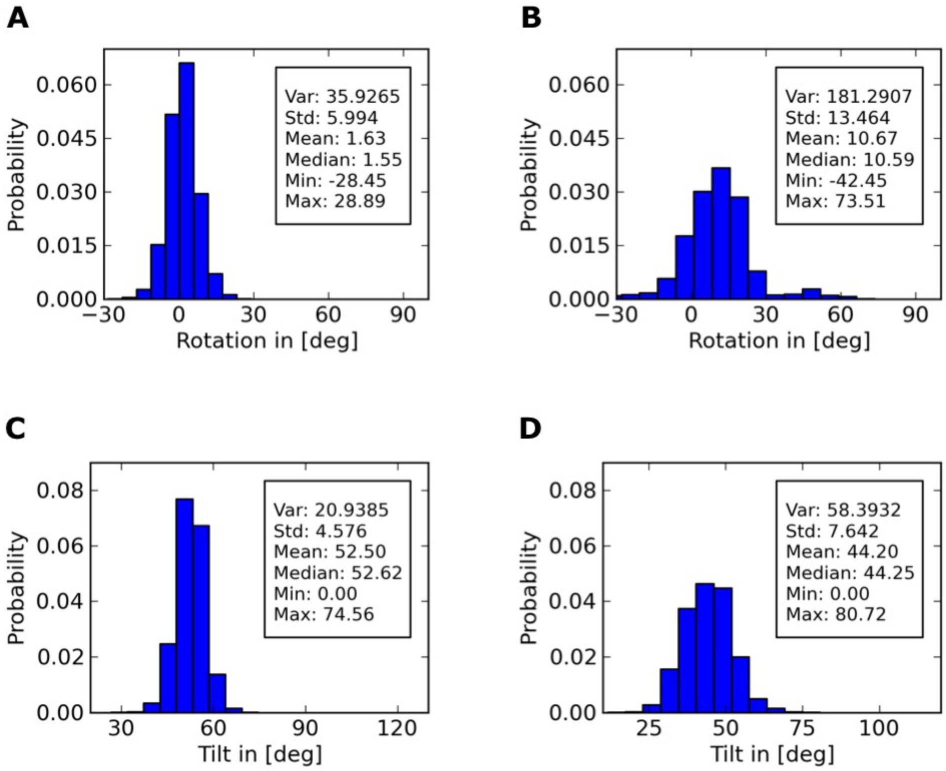

Fig E. Mobility of GαCT in β_2AR*•GsαCT and RhR*•GtαCT measured by rotation and tilt. Histogram for the rotation around the helix axis of **(A)** GsαCT and **(B)** GtαCT. Histogram of the helix tilt motion perpendicular to the membrane plane of **(C)** GsαCT and **(D)** GtαCT. The analyses are based on 10 x 200 ns simulations each (see Fig L). For definition of helix axis and tilt see *Calculation of helix axis, helix tilt and rotation*. The peaks of the tilt angle distribution are clearly different, although the standard deviations overlap.

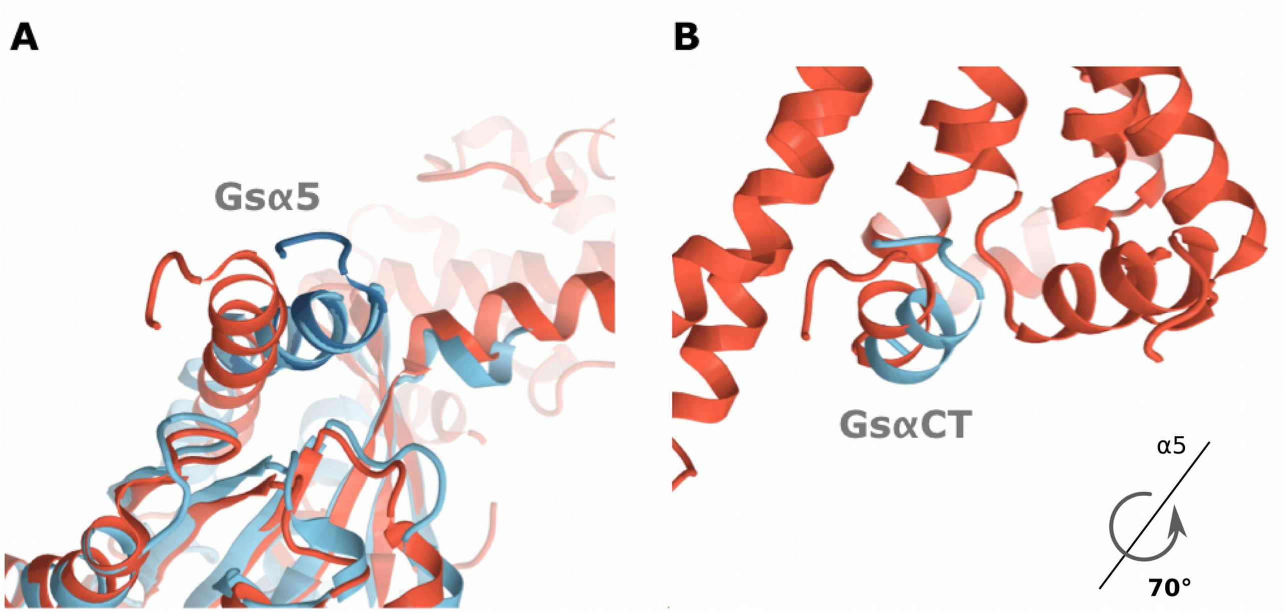

Fig F. (A) α5 helix-switch in transition from Gs[GDP] to β_2AR*•Gs[empty] and (B) switch-like movement of GsαCT in the cytoplasmic crevice of β_2AR*. Comparison of the α5 helix-switch in G with orientations of GsαCT in the cytoplasmic crevice of R* reveals a very similar rotation of about 60°. The nucleotide bound Gs (Gsα[GTPγS], PDB entry: 1AZT, colored in light blue) was superimposed to β_2AR*•Gsα[empty] (PDB entry: 3SN6, colored in red) by the Ras domain, excluding α5. The unresolved C-terminal reverse turn in Gsα[GTPγS] was modeled from superimposing the α5 helix of β_2AR*•Gsα[empty] (dark blue). The orientation of GsαCT in a putative β_2AR*•Gs[GDP] intermediate (colored in light blue) was obtained from flexible docking analysis (Fig G and *Flexible docking analysis*) and its orientation in the X-ray (colored in red) was derived from PDB entry 3SN6. Superimposition with Giα[GDP] (PDB entry 1GP2) instead of Gsα[GTPγS] reveals a very similar rotation of α5.

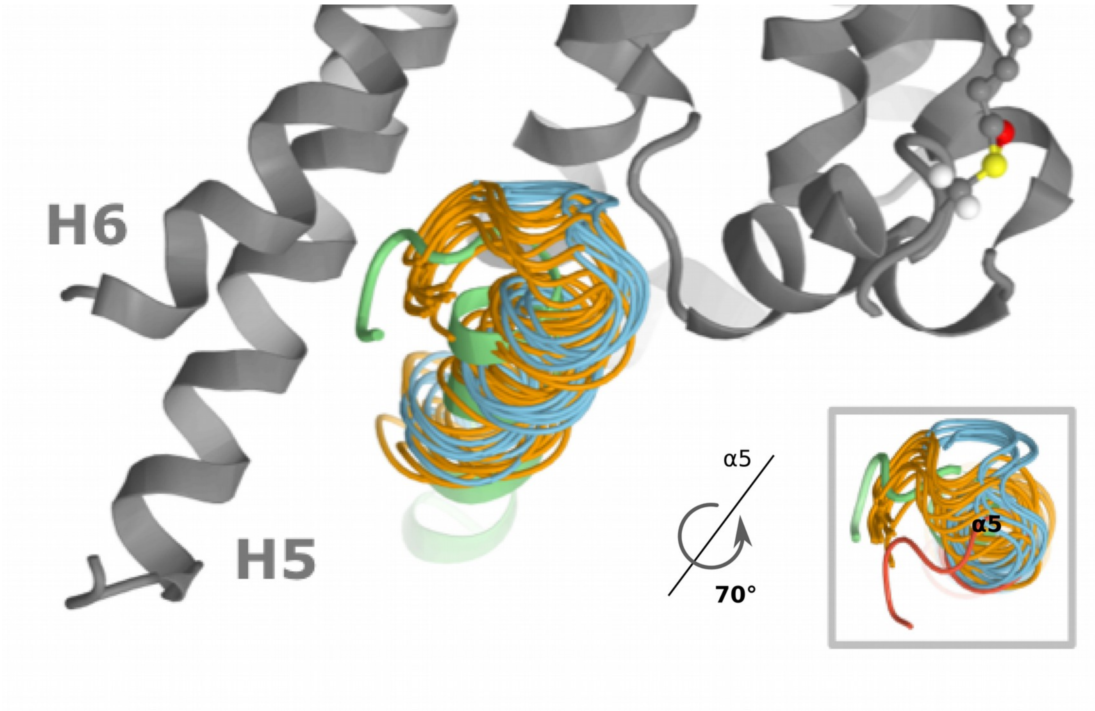

Fig G. Clusters of docking poses of 15-mer GsαCT to β2AR*. The largest cluster (orange) coincides with the position and orientation of GsαCT in the X-ray structure. However, in agreement with the MD simulations of β2AR*•GsαCT (Fig B) GsαCT is shifted away from TM3 as compared to its position in the β2AR*•Gs[empty] complex. In the blue cluster (third largest cluster) GsαCT is rotated by 60° compared to the X-ray state and represents the position in a putative R*•G[GDP] intermediate. The inset shows the view along the helix axis highlighting the difference between both states. The orientation of GsαCT from the second largest cluster (representative structure shown in red in the inset) is neither compatible with the X-ray state nor with the R*•G[GDP] intermediate, because it over twists the rotation of GsαCT. Hypothetically, this position refers to one of the intermediate dissociation states observed by single particle EM of the β2AR*•Gs complex [35].

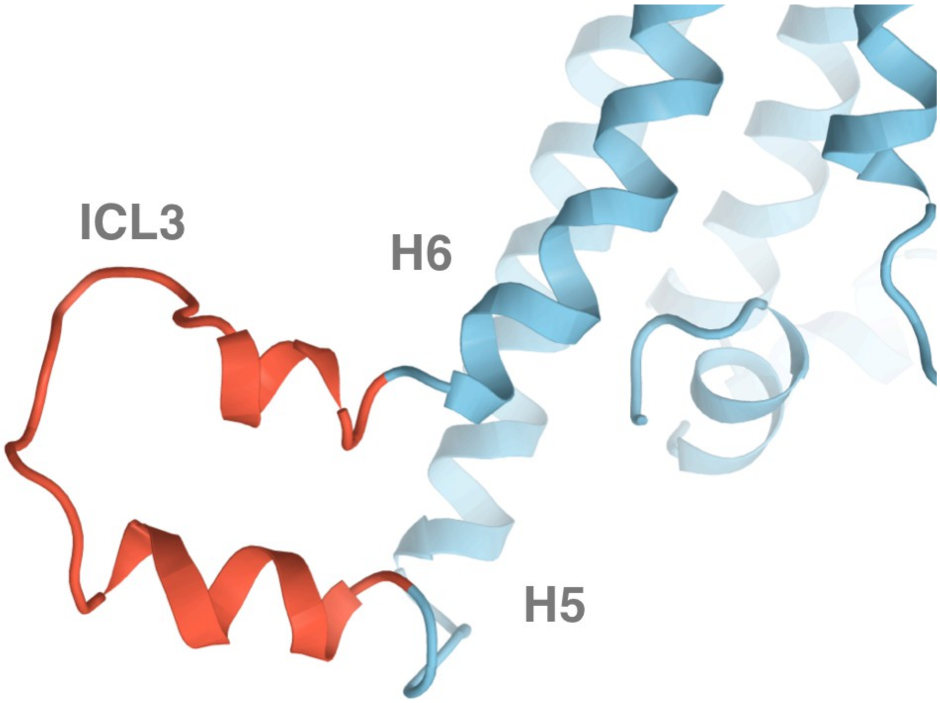

Fig H. Structure of modeled ICL3 of β_2AR*. The protein loop was modeled with the program SuperLooper [7] which utilizes existing loops linking transmembrane helices from structures in the PDB (www.rcsb.org). See also *Preparations of active receptor and GαCT structures and complexes*.

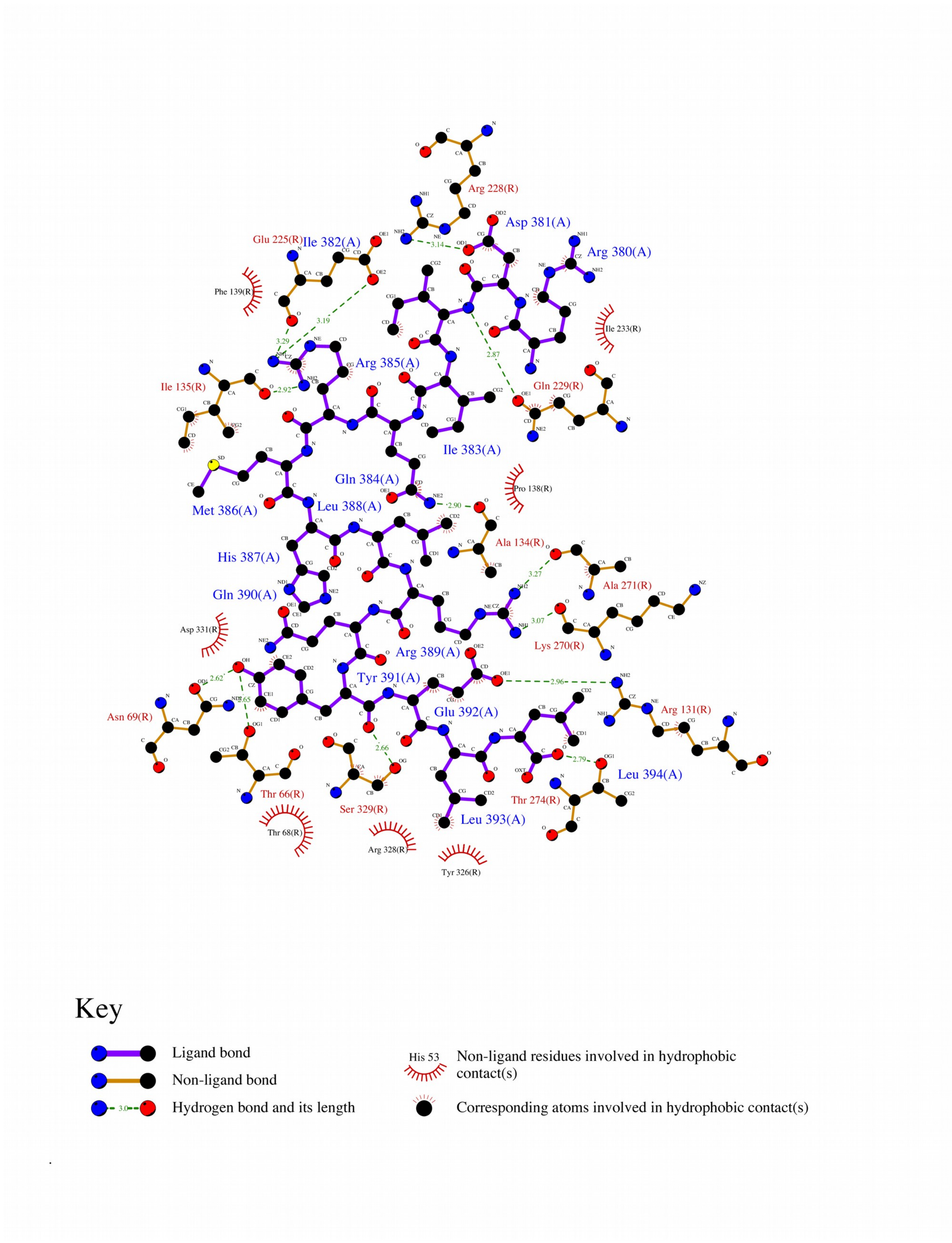

Fig Ia. Interactions between β$_2$AR* and GsαCT in the putative R*•G[GDP] intermediate. Potential hydrogen bonds and van der Waals contacts were analyzed using the programs HBPLUS [36] and LIGPLOT [37]. Residues with closest distances less than 4 Å are considered to be in van der Waals contact.

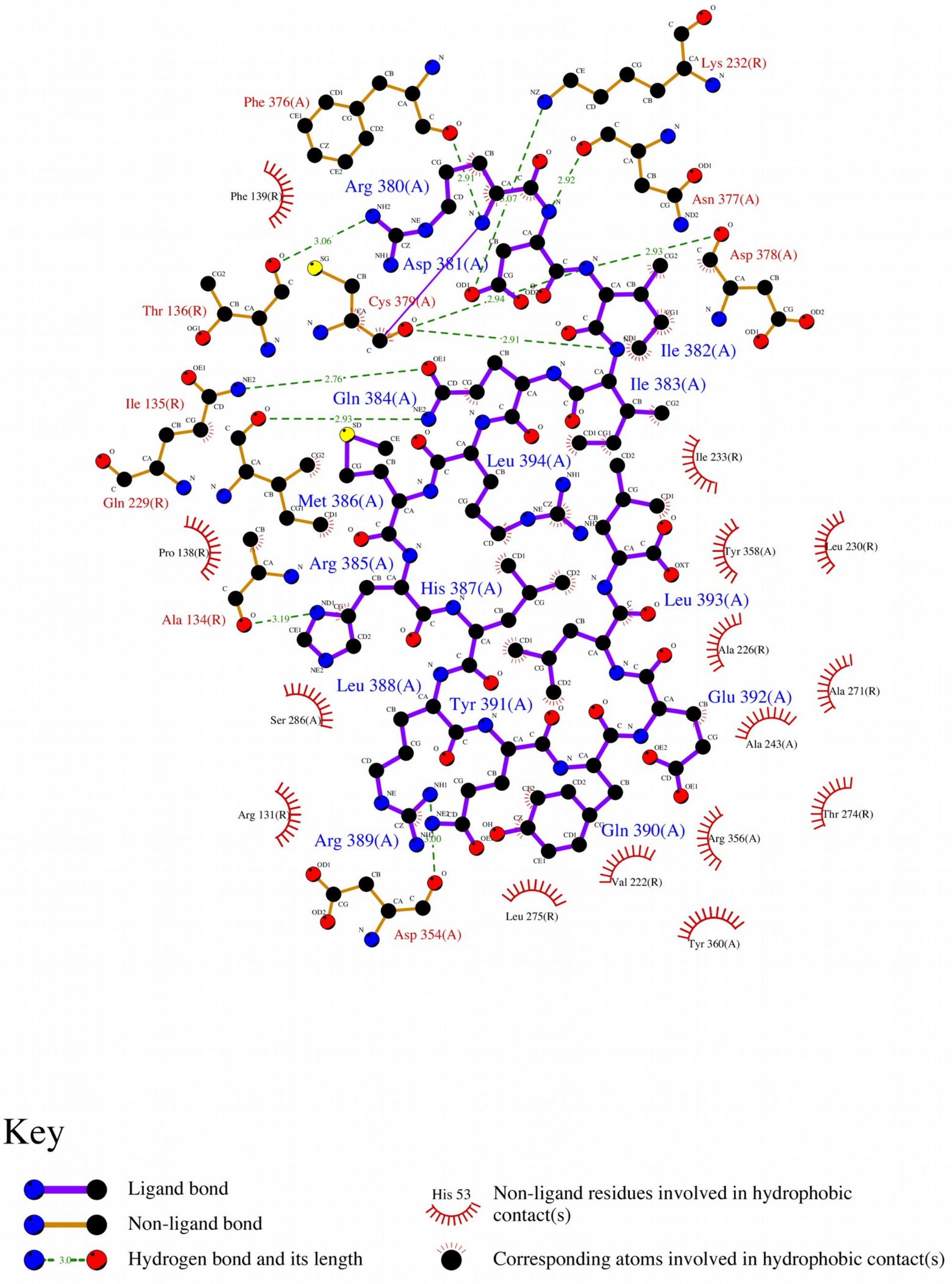

Fig Ib. Interactions between β_2AR* and GsαCT in the position and orientation observed in the X-ray structures (PDB entry 3SN6). Potential hydrogen bonds and van der Waals contacts were analyzed using the programs HBPLUS [36] and LIGPLOT [37]. Residues with closest distances less than 4 Å are considered to be in van der Waals contact.

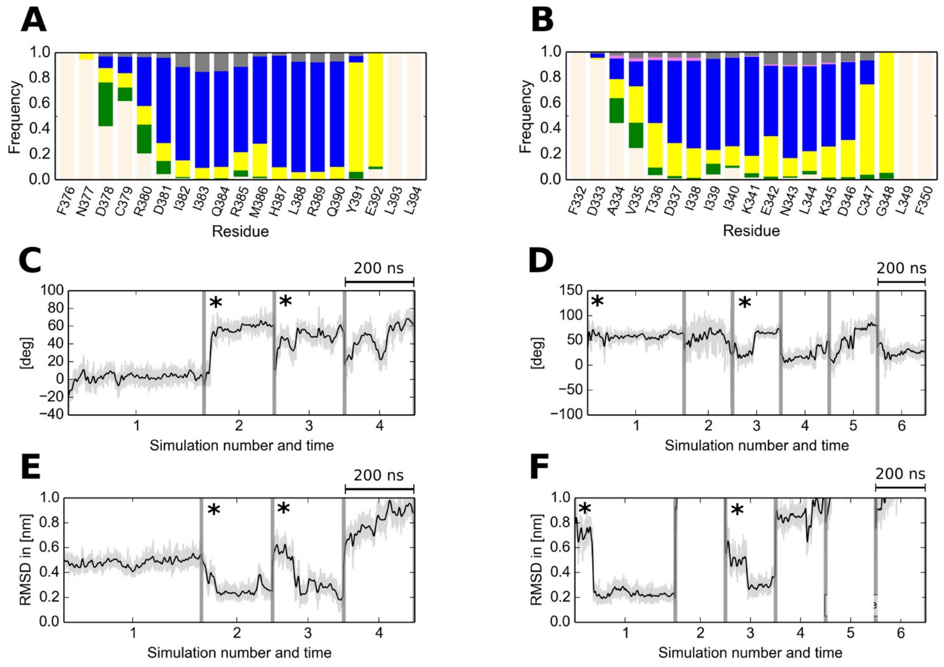

Fig J. Secondary structure analysis and transitions from the intermediate position of 19-mer GαCT to the final β_2AR*•GsαCT (A, C, E) and RhR*•GsαCT complexes (B, D, E). (A, B) Starting from the intermediate GαCT positions obtained by flexible docking, analysis with the program DSSP of the combined 19-mer GαCT simulations shows stable helical core regions (blue), but helix-coil transitions at the N-terminus (pale orange). See also Fig A. The observed rotation and RMSD of GαCT are given for the individual simulations. (C, D) Change in rotation of (C) GsαCT or (D) GtαCT around its helix axis. (E, F) Backbone-RMSD of (E) GsαCT or (F) GtαCT relative to the position in the X-ray structure. In simulation marked with a *, the helix-switch occurred, i.e. a rotation of about 60° and a decrease in RMSD below 4 Å was observed.

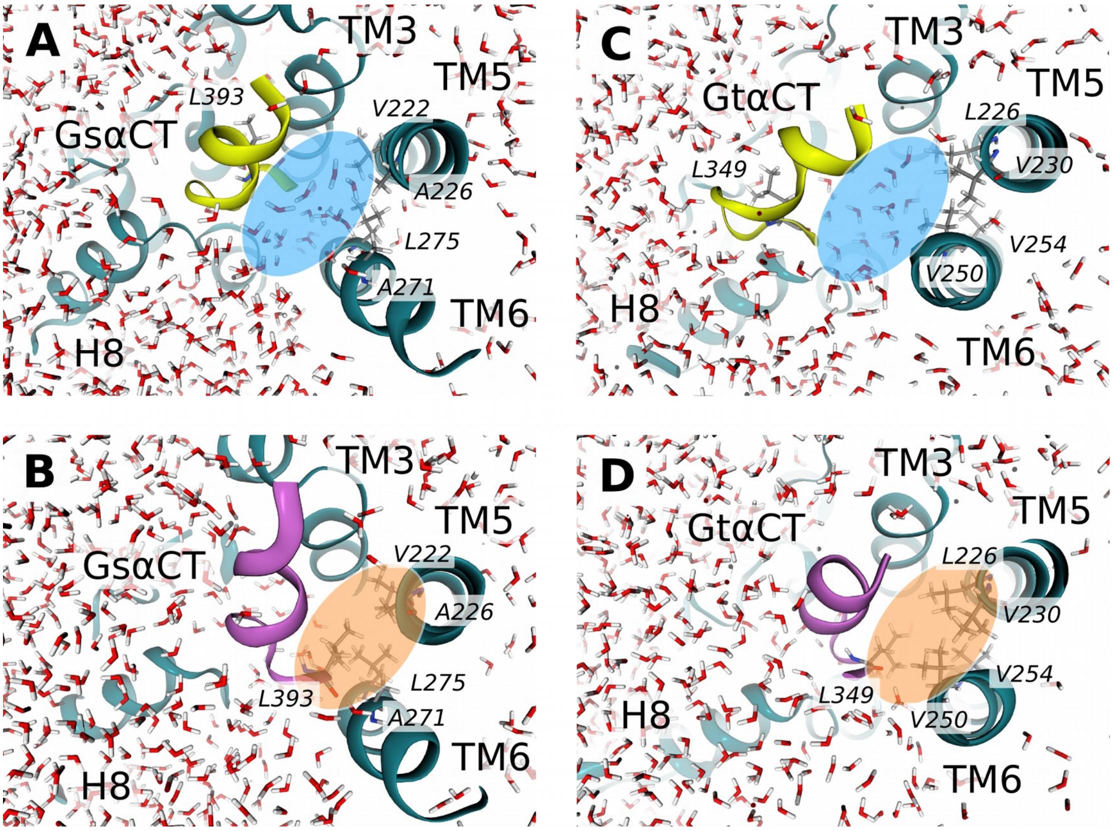

Fig K. Water expulsion accompanying the helix-switch. In the intermediate state of β_2AR*•GsαCT (A) and RhR*•GtαCT (C) water molecules (blue ellipsis) are located between GαCT and TM5 and 6 of R*. During the helix-switch and formation of the final β_2AR*•GsαCT (B) and RhR*•GtαCT (D) complex these water molecules are displaced in favor of hydrophobic interactions (orange ellipsis) between GαCT and TM5 and 6 of R*. For time-series data of the water expulsion see Fig R and for hydrophobic patch formation see Fig Q.

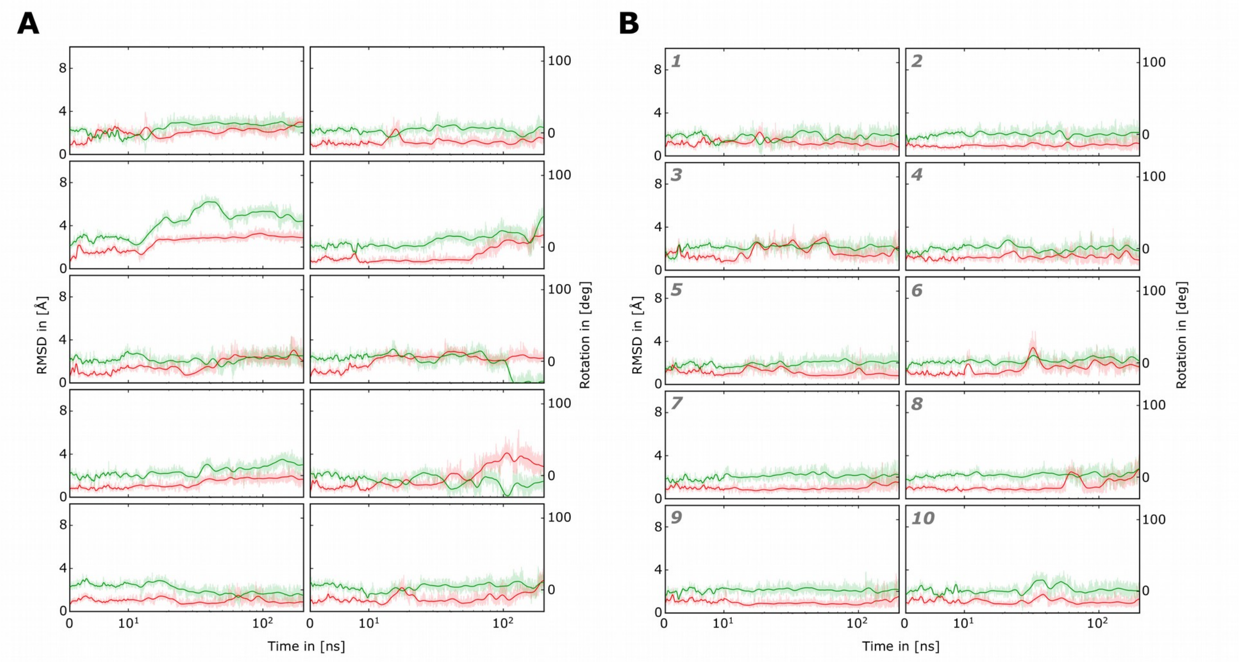

Fig L. Mobility of GαCT in (A) β_2AR*•GsαCT and (B) RhR*•GtαCT measured by rotation and backbone-RMSD. The rotation of GαCT around its helix axis during ten 200 ns simulations, starting from the GαCT position of the X-ray structure, is depicted in green. The peptide RMSD (red) describes the movement of GαCT relative to its position in the co-crystal. For definitions of helix axis, rotation and peptide RMSD see *Calculation of helix axis, helix tilt and rotation*. The plots are linear for the first 10 ns and logarithmic for the remaining time.

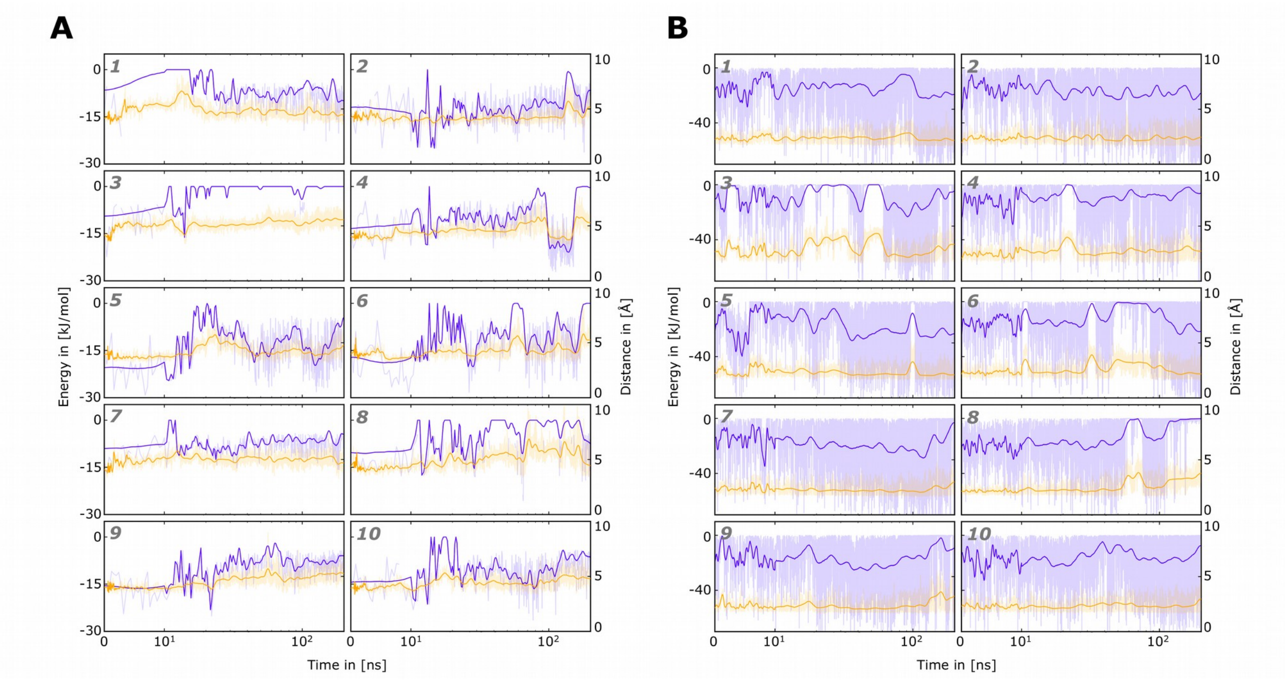

Fig M. Distance and interaction energy of $R^{3.50}$ in β_2AR*•GsαCT and RhR*•GtαCT. **(A)** Distance (yellow) and cation-π interaction energy (blue) between the center of the phenylring of Y391 in GsαCT and $R131^{3.50}$ during ten 200 ns simulations, starting from the GαCT position of the X-ray structure. For clarity, only energies < −4.1 kJ/mol are shown. **(B)** Distance (yellow) and hydrogen bond energy (blue) between the carbonyl oxygen of C348 in GtαCT and $R135^{3.50}$ during ten 200 ns simulations. The plots are linear for the first 10 ns and logarithmic for the remaining time.

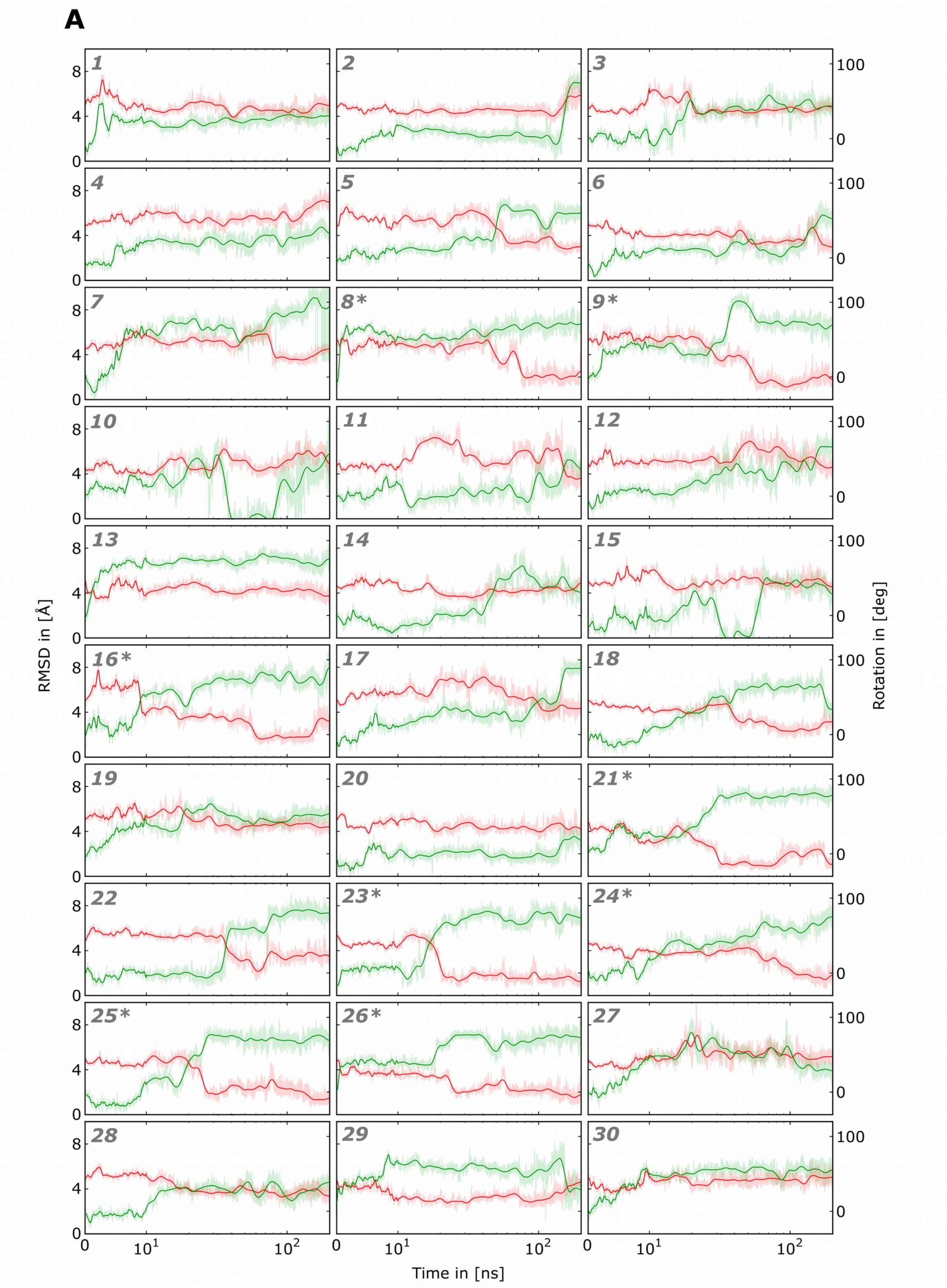

Fig Na. Mobility of GsαCT in β$_2$AR*•GsαCT measured by rotation and backbone-RMSD. The rotation around the helix axis during thirty 200 ns simulations is depicted in green. The peptide RMSD (red) describes the movement of GsαCT relative to its position in the X-ray structure. For definitions of helix axis, rotation and peptide RMSD see *Calculation of helix axis, helix tilt and rotation*. Simulations in which a switch event occurs are marked with a star (*) in the upper left corner. The plots are linear for the first 10 ns and logarithmic for the remaining time.

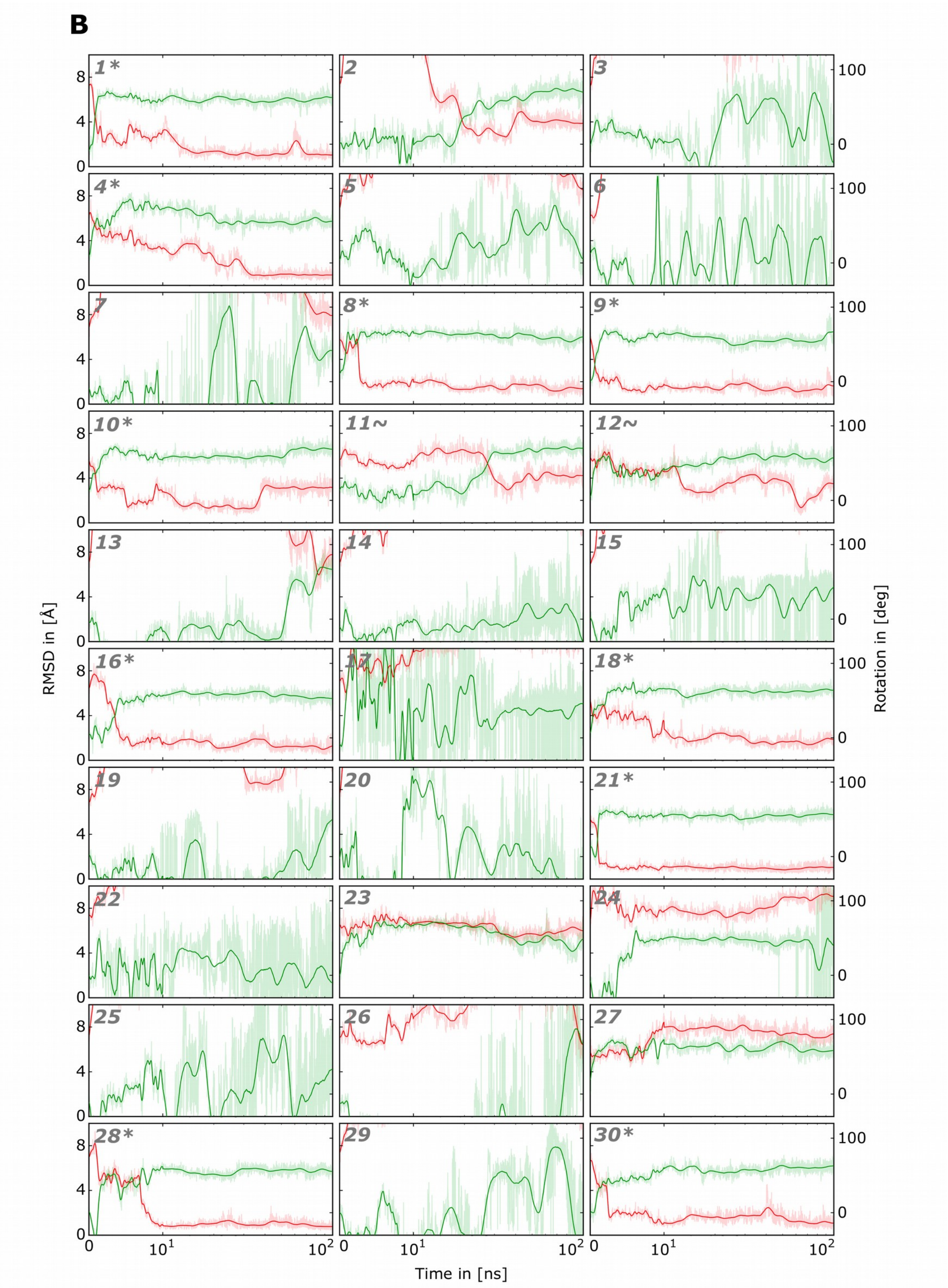

Fig Nb. Mobility of RhR*•GtαCT measured by rotation and backbone-RMSD. The rotation around the helix axis during thirty 100 ns simulations is depicted in green. The peptide RMSD (red) describes the movement of GtαCT relative to its position in the crystal structure. For definitions of helix axis, rotation and peptide RMSD see *Calculation of helix axis, helix tilt and rotation* in *S1 Protocol*. Simulations in which a switch event occurs are marked with a star (*) in the upper left corner. Simulations in which GtαCT adopts a stable binding mode but does not switch are marked with a tilde (~) in the upper left corner. The plots are linear for the first 10 ns (dashed grey line) and logarithmic for the remaining time.

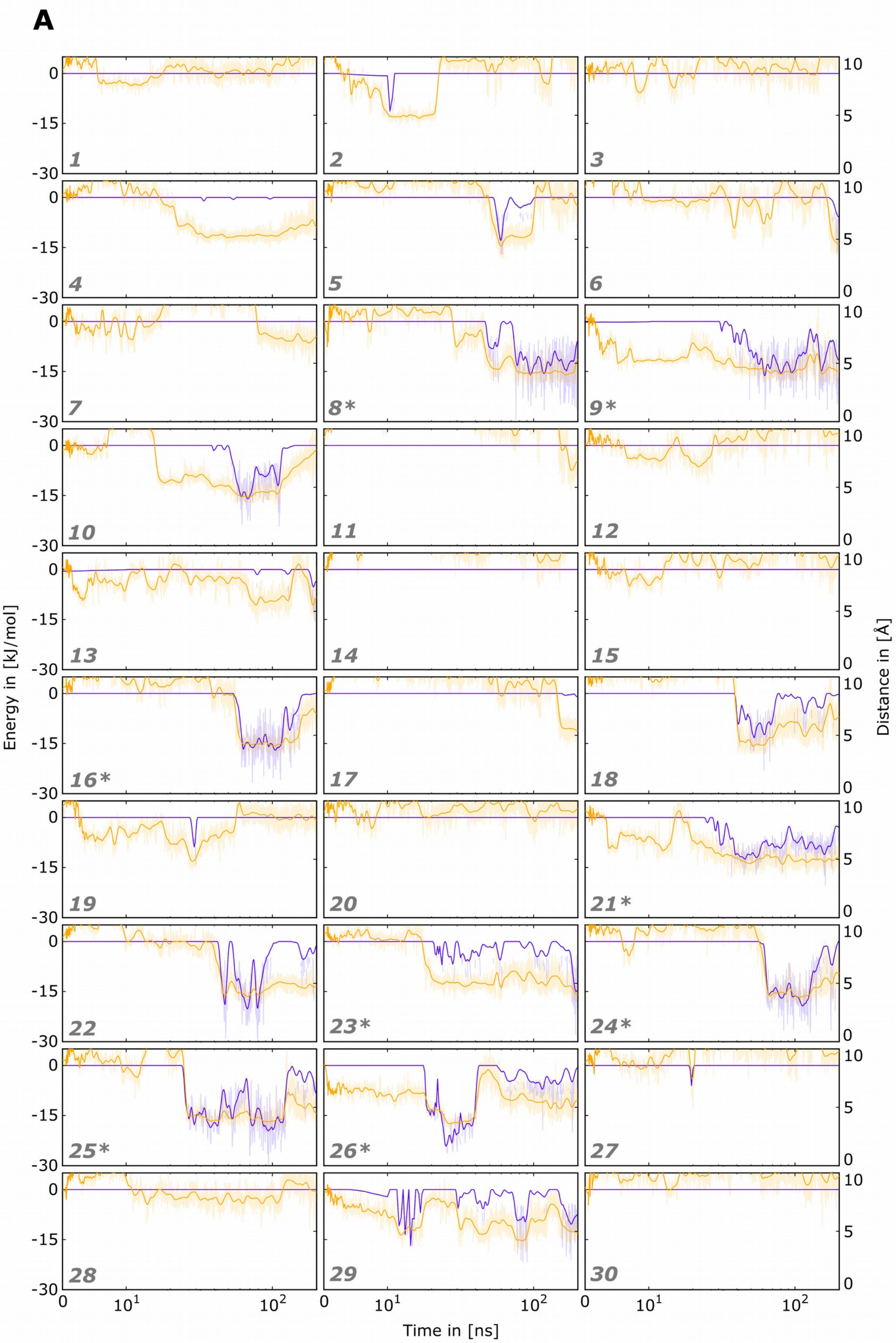

Fig Oa. Distance and interaction energies of R131$^{3.50}$ in β_2AR*•GsαCT.

Distance (yellow) and cation-π interaction energy (blue) between the centre of the phenyl ring of Y391 in GsαCT and R131$^{3.50}$ during ten 200 ns simulations. For clarity, only energies < −4.1 kJ/mol are shown. Simulations in which a switch event occurs are marked with a star (*) in the lower left corner. The plots are linear for the first 10 ns (dashed grey line) and logarithmic for the remaining time.

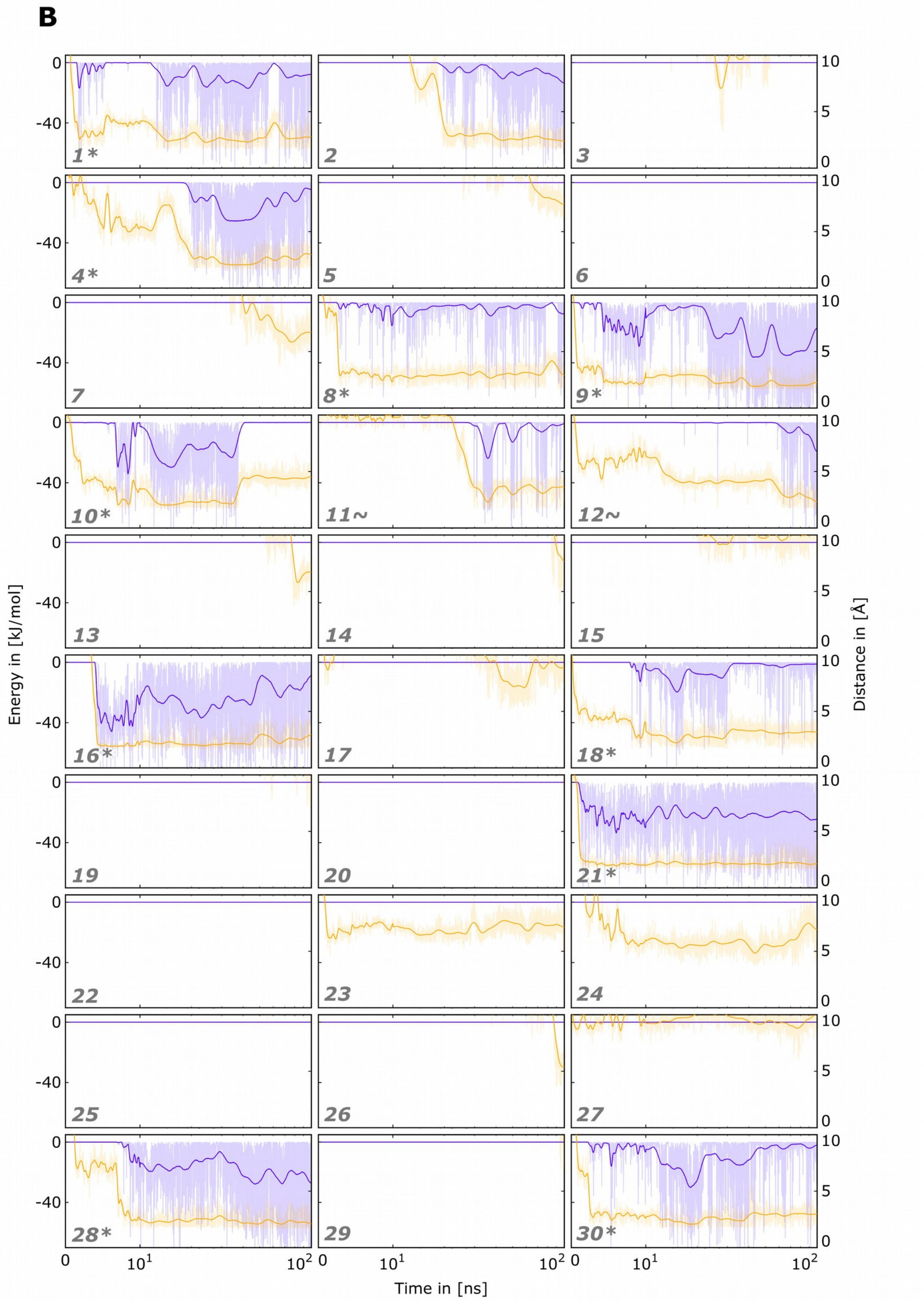

Fig Ob. Distance and interaction energies of R135[3.50] RhR*•GtαCT. Distance (yellow) and hydrogen bond energy (blue) between carbonyl oxygen of C348 in GtαCT and R135[3.50] during thirty 100 ns simulations. Simulations in which a switch event occurs are marked with a star (*) in the lower left corner. Simulations in which GtαCT adopts a stable binding mode but does not switch are marked with a tilde (~) in the lower left corner. The plots are linear for the first 10 ns (dashed grey line) and logarithmic for the remaining time.

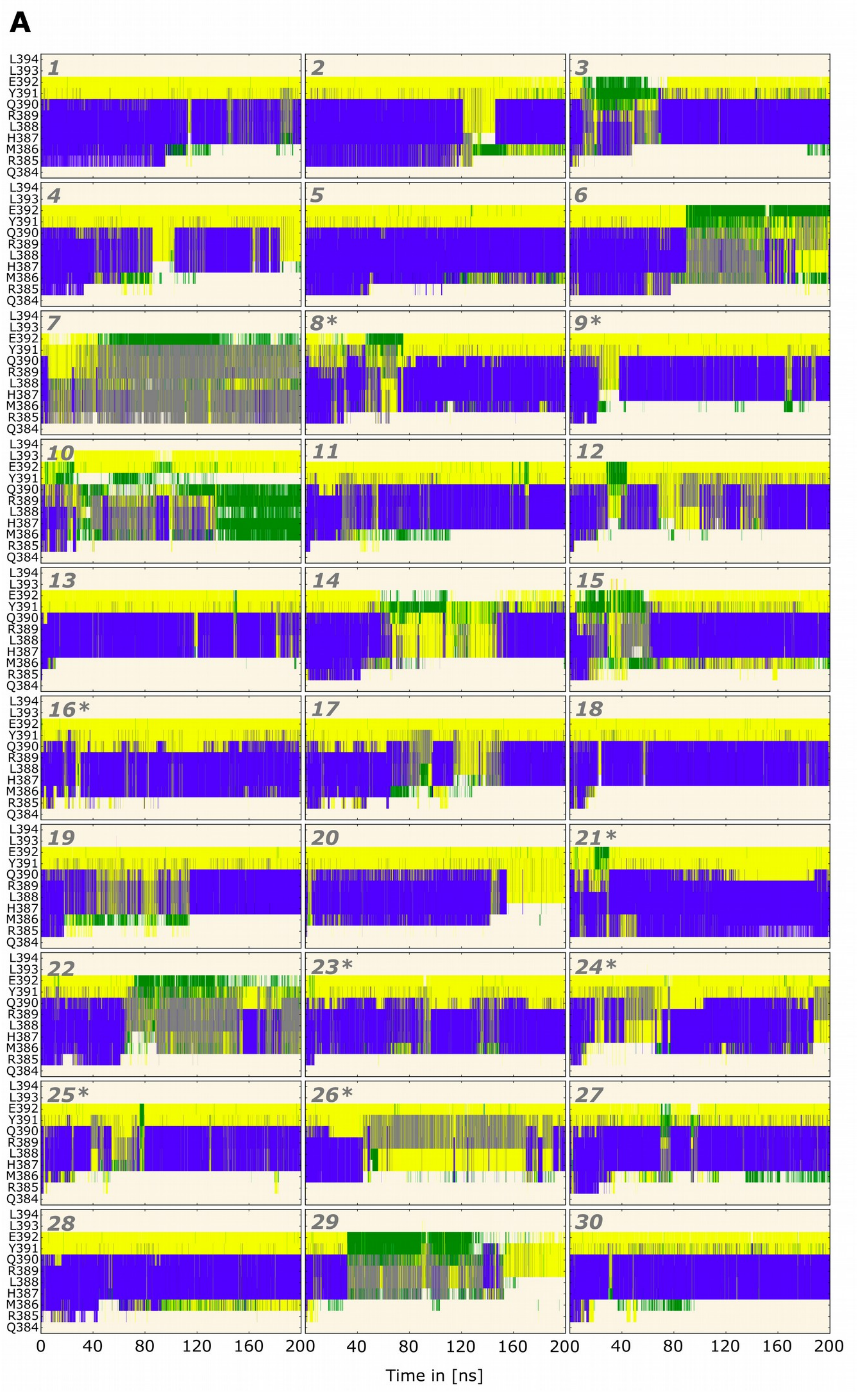

Fig Pa. Secondary structure analysis of GsαCT in β$_2$AR*•GsαCT. For each GsαCT residue the secondary structure according to the program DSSP is shown over time (blue: α-helix, 3_{10}-helix: grey, turn: yellow, green: bend, coil: pale orange. Simulations in which a switch event occurs are marked with a grey star (*) in the upper left corner.

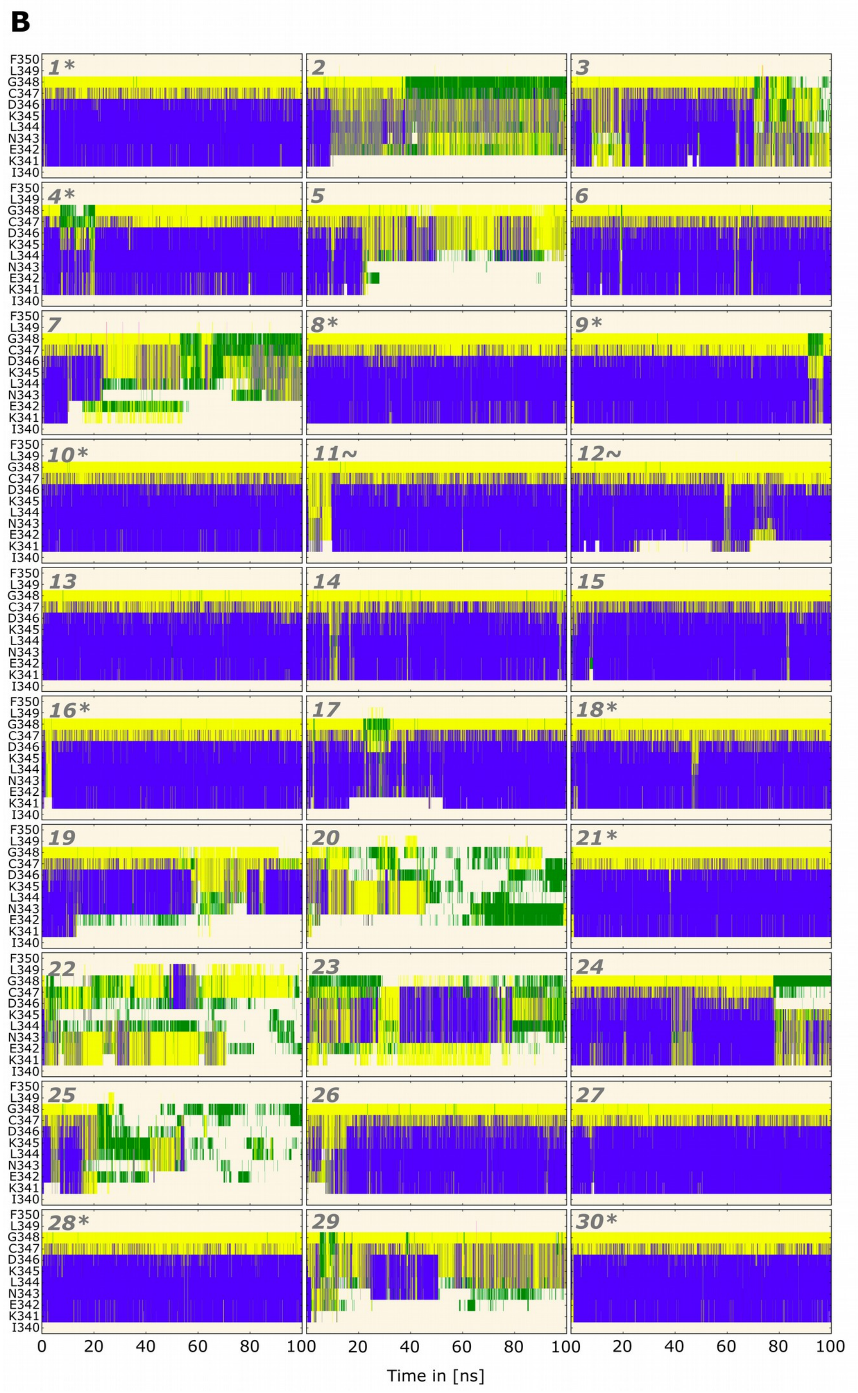

Fig Pb. Secondary structure analysis of GtαCT in RhR*•GtαCT. For each GtαCT residue the secondary structure according to the program DSSP is shown over time (blue: α-helix, 3_{10}-helix: grey, turn: yellow, green: bend, coil: pale orange. Simulations in which a switch event occurs are marked with a grey star (*) in the upper left corner.

Fig Qa. Hydrophobic patch between GsαCT and β$_2$AR*. The minimal distances between L393 of GsαCT and V222 (black), A226 (red), A271 (blue) and L275 (green), respectively, of TM5 and 6 of β$_2$AR are plotted over time. The plots are linear for the first 10 ns (dashed grey line) and logarithmic for the remaining time.

Fig Qb. Hydrophobic patch between GtαCT and RhR*. The minimal distances between L349 of GtαCT and L226 (black), V230 (red), V250 (blue) and V254 (green), respectively, of TM5 and 6 of RhR* are plotted over time. The plots are linear for the first 10 ns (dashed grey line) and logarithmic for the remaining time.

Fig Ra. Hydration status of the hydrophobic patch of β_2AR* at GsαCT interface. The number of contacts < 2.5 Å between any water molecule and V222, A226, A271 and L275 of TM5 and 6 of β_2AR are plotted over time. Simulations in which a switch event occurs are marked with a grey star (*). The plots are linear for the first 10 ns (dashed grey line) and logarithmic for the remaining time.

Fig Rb. Hydration status of the hydrophobic patch of RhR* at GtαCT interface. The number of contacts < 2.5 Å between any water molecule and L226, V230, V250 and V254 of TM5 and 6 of RhR* are plotted over time. Simulations in which a switch event occurs are marked with a grey star (*). The plots are linear for the first 10 ns (dashed grey line) and logarithmic for the remaining time.

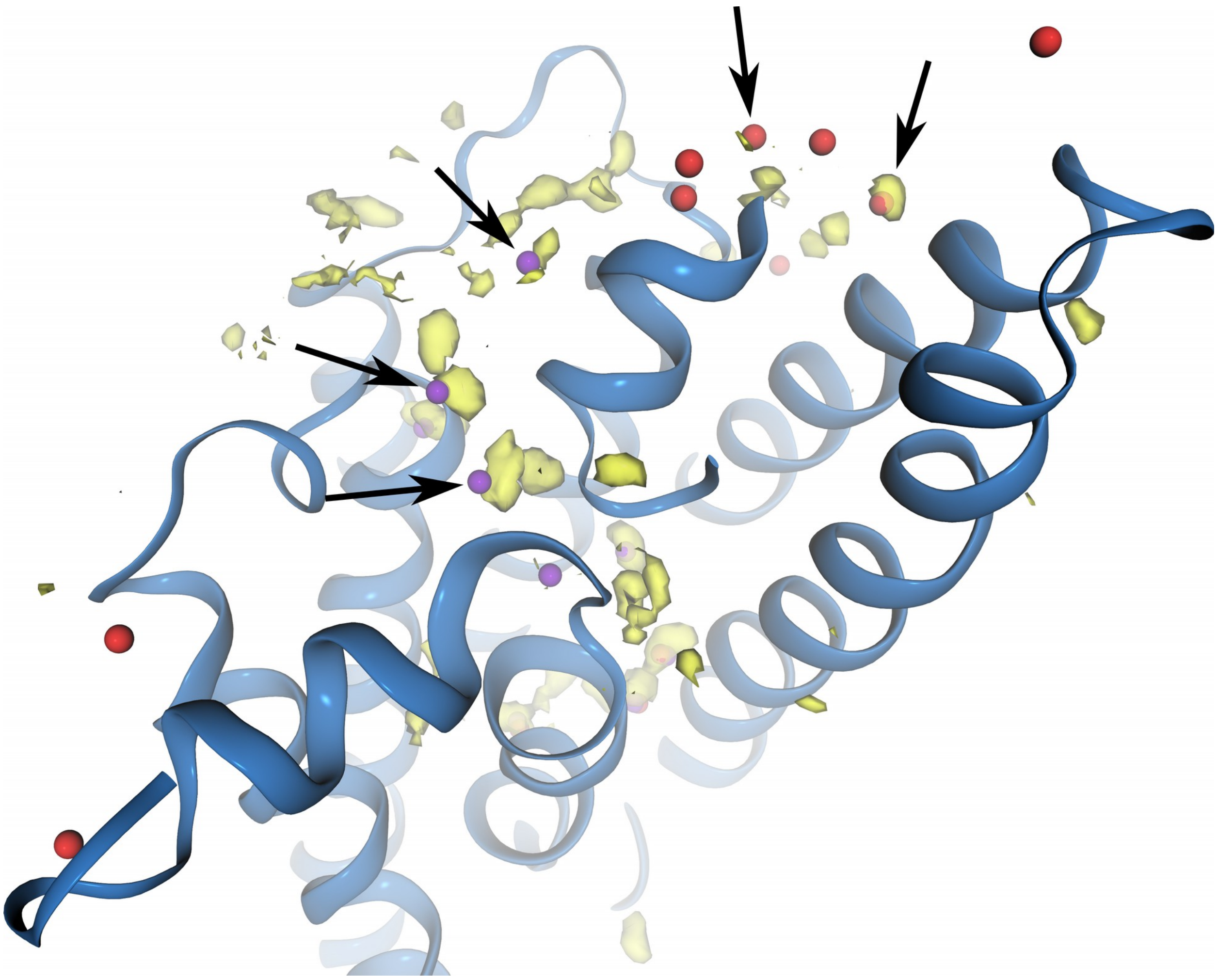

Fig S. Water molecules at the RhR* GtαCT interface observed after a "helix-switch" in MD simulations compared to waters resolved by X-ray structure analysis. Regions of high water density (yellow isosurface) observed in simulation 1 of RhR*•GtαCT, in which GtαCT switches, are compared to water molecules resolved in two X-ray structures of RhR*•GtαCT (PDB entries 3dqb, red, and 2x72, purple). Black arrows indicate places where high density water regions coincide with crystallographic water molecules. A snapshot of the RhR*•GtαCT complex (blue cartoon) from the simulation is shown for context. Note that in the snapshot the peptide has already switched from the intermediate position to the position resolved in the X-ray structures of RhR*•GtαCT. This suggests that the observed water molecule density is not biased by the starting structure of the simulation but arises independently after the helix switch. The water molecule density was calculated with the GROMACS tool g_spatial using a bin width of 0.05 nm.

References

1. Rasmussen SGF, Devree BT, Zou Y, Kruse AC, Chung KY, Kobilka TS, et al. Crystal structure of the β(2) adrenergic receptor-Gs protein complex. Nature. 1] Department of Molecular and Cellular Physiology, Stanford University School of Medicine, Stanford, California 94305, USA [2] Department of Neuroscience and Pharmacology, The Panum Institute, University of Copenhagen, 2200 Copenhagen N, Denmark [3].: Nature Publishing Group; 2011;450: 383–387. Available: http://eutils.ncbi.nlm.nih.gov/entrez/eutils/elink.fcgi?dbfrom=pubmed&id=21772288&retmode=ref&cmd=prlinks

2. Scheerer P, Park JH, Hildebrand PW, Kim YJ, Krauss N, Choe H-W, et al. Crystal structure of opsin in its G-protein-interacting conformation. Nature. Institut für Medizinische Physik und Biophysik (CC2), Charité - Universitätsmedizin Berlin, Chariteplatz 1, D-10117 Berlin, Germany.: Nature Publishing Group; 2008;455: 497–502. Available: http://www.nature.com/nature/journal/v455/n7212/abs/nature07330.html

3. Chen J, Makino CL, Peachey NS, Baylor DA, Simon MI. Mechanisms of rhodopsin inactivation in vivo as revealed by a COOH-terminal truncation mutant. Science. 1995;267: 374–7. Available: http://www.ncbi.nlm.nih.gov/pubmed/7824934

4. Dunbrack RL, Cohen FE. Bayesian statistical analysis of protein side-chain rotamer preferences. Protein Sci. 1997;6: 1661–81. doi:10.1002/pro.5560060807

5. Rasmussen SGF, Choi H-J, Fung JJ, Pardon E, Casarosa P, Chae PS, et al. Structure of a nanobody-stabilized active state of the β(2) adrenoceptor. Nature. Department of Molecular and Cellular Physiology, Stanford University School of Medicine, 279 Campus Drive, Stanford, California 94305, USA.: Nature Publishing Group; 2011;469: 175–180. doi:10.1038/nature09648

6. Rubenstein RC, Wong SK, Ross EM. The hydrophobic tryptic core of the beta-adrenergic receptor retains Gs regulatory activity in response to agonists and thiols. J Biol Chem. 1987;262: 16655–16662. Available: http://www.jbc.org/cgi/content/abstract/262/34/16655

7. Hildebrand PW, Goede A, Bauer RA, Gruening B, Ismer J, Michalsky E, et al. SuperLooper--a prediction server for the modeling of loops in globular and membrane proteins. Nucleic Acids Res. Institute of Medical Physics and Biophysics, Charité, University of Medicine, Berlin, Germany. peter.hildebrand@charite.de: Oxford University Press; 2009;37: W571–4. doi:10.1093/nar/gkp338

8. Verdonk ML, Cole JC, Hartshorn MJ, Murray CW, Taylor RD. Improved protein-ligand docking using GOLD. Proteins. 2003;52: 609–23. Available: http://www.ncbi.nlm.nih.gov/pubmed/12910460

9. Rath P, DeCaluwe L, Bovee-Geurts P. Fourier transform infrared difference spectroscopy of rhodopsin mutants: light activation of rhodopsin causes hydrogen-bonding change in residue aspartic acid-83. Biochemistry. 1993;32. Available: http://pubs.acs.org/doi/abs/10.1021/bi00090a001

10. Fahmy K, Jager F, Beck M, Zvyaga TA, Sakmar TP, Siebert F. Protonation states of membrane-embedded carboxylic acid groups in rhodopsin and metarhodopsin II : A Fourier-transform infrared spectroscopy study of site-directed mutants. Proc Natl Acad Sci USA. Howard Hughes Medical Institute, Rockefeller University, New York, NY 10021.; 1993;90: 10206–10210. Available: http://www.ncbi.nlm.nih.gov/pmc/articles/PMC47743/

11. Jaeger F, Fahmy K, Sakmar T. Identification of glutamic acid 113 as the Schiff base proton acceptor in the metarhodopsin II photointermediate of rhodopsin. Biochemistry. 1994;33: 10878–10882. Available: http://pubs.acs.org/doi/abs/10.1021/bi00202a005

12. Fahmy K, Sakmar T, Siebert F. Transducin-dependent protonation of glutamic acid 134 in rhodopsin. Biochemistry. 2000;39: 10607–10612. Available: http://pubs.acs.org/doi/abs/10.1021/bi000912d

13. Dror RO, Arlow DH, Borhani DW, Jensen MØ, Piana S, Shaw DE. Identification of two distinct inactive conformations of the beta2-adrenergic receptor reconciles structural and biochemical observations. Proc Natl Acad Sci U S A. D. E. Shaw Research, New York, NY 10036, USA.; 2009;106: 4689–4694. Available: http://eutils.ncbi.nlm.nih.gov/entrez/eutils/elink.fcgi?dbfrom=pubmed&id=19258456&retmode=ref&cmd=prlinks

14. Zhang L, Hermans J. Hydrophilicity of cavities in proteins. Proteins. Department of Biochemistry and Biophysics, University of North Carolina, Chapel Hill 27599-7260, USA.; 1996;24: 433–8. doi:10.1002/(SICI)1097-0134(199604)24:4<433::AID-PROT3>3.0.CO;2-F

15. Hildebrand PW, Scheerer P, Park JH, Choe H-W, Piechnick R, Ernst OP, et al. A Ligand Channel through the G Protein Coupled Receptor Opsin. PLoS One. Institut für Medizinische Physik und Biophysik, Charité-Universitätsmedizin Berlin, Berlin, Germany. peter.hildebrand@charite.de; 2009;4: e4382. Available: http://www.plosone.org/article/info:doi/10.1371/journal.pone.0004382

16. Scheerer P, Heck M, Goede A, Park J, Choe H, Ernst OP, et al. Structural and kinetic modeling of an activating helix switch in the rhodopsin-transducin interface. Proc Natl Acad Sci U S A. Institut für Medizinische Physik und Biophysik (CC2) and.;

2009;106: 10660–10665. Available: http://www.pnas.org/content/106/26/10660.abstract

17. Shan J, Weinstein H, Mehler EL. Probing the structural determinants for the function of intracellular loop 2 in structurally cognate G-protein-coupled receptors. Biochemistry. American Chemical Society; 2010;49: 10691–701. doi:10.1021/bi100580s

18. Hess B, Kutzner C, van der Spoel D, Lindahl E. GROMACS 4: Algorithms for Highly Efficient, Load-Balanced, and Scalable Molecular Simulation. J Chem Theory Comput. American Chemical Society; 2008;4: 435–447. doi:10.1021/ct700301q

19. Wolf MG, Hoefling M, Aponte-Santamaría C, Grubmüller H, Groenhof G. g_membed: Efficient insertion of a membrane protein into an equilibrated lipid bilayer with minimal perturbation. J Comput Chem. 2010;31: 2169–74. doi:10.1002/jcc.21507

20. Berger O, Edholm O. Molecular dynamics simulations of a fluid bilayer of dipalmitoylphosphatidylcholine at full hydration, constant pressure, and constant temperature. Biophys J. 1997;72: 2002–2013. Available: http://www.sciencedirect.com/science/article/pii/S0006349597788453

21. Berendsen HJC, Grigera JR, Straatsma TP. The Missing Term in Effective Pair Potentials. J Phys Chem. Inst Fis Liquidos & Sistemas Biol,Ra-1900 La Plata,Argentina; 1987;91: 6269–6271. Available: http://links.isiglobalnet2.com/gateway/Gateway.cgi?GWVersion=2&SrcAuth=mekentosj&SrcApp=Papers&DestLinkType=FullRecord&DestApp=WOS&KeyUT=A1987K994100038

22. Lindorff-Larsen K, Piana S, Palmo K, Maragakis P, Klepeis JL, Dror RO, et al. Improved side-chain torsion potentials for the Amber ff99SB protein force field. Proteins. D. E. Shaw Research, New York, New York 10036, USA.; 2010;78: 1950–1958. Available: http://sfx.mpg.de/sfx_local?id=doi:10.1002/prot.22711

23. Schüttelkopf AW, van Aalten DMF. PRODRG: a tool for high-throughput crystallography of protein-ligand complexes. Acta Crystallogr D Biol Crystallogr. Division of Biological Chemistry and Molecular Microbiology, Wellcome Trust Biocentre, School of Life Sciences, University of Dundee, Dow Street, DD1 5EH, Scotland.; 2004;60: 1355–63. doi:10.1107/S0907444904011679

24. Wang J, Wang W, Kollman P a, Case D a. Automatic atom type and bond type perception in molecular mechanical calculations. J Mol Graph Model. 2006;25: 247–60. doi:10.1016/j.jmgm.2005.12.005

25. Wang J, Wolf RRM, Caldwell JJWJ, Kollman PA, Case DA. Development and testing of a general amber force field. J Comput Chem. 2004;25: 1157–1174. Available: http://onlinelibrary.wiley.com/doi/10.1002/jcc.20035/full

26. Sousa da Silva AW, Vranken WF, Sousa Da Silva, A. W. & Vranken W. ACPYPE - AnteChamber PYthon Parser interfacE. In: BMC Res Notes [Internet]. 23 Jul 2012 p. 367. doi:10.1186/1756-0500-5-367

27. Hess B, Bekker H, Berendsen HJC, Fraaije JGEM. LINCS: A linear constraint solver for molecular simulations. J Comput Chem. 1997;18: 1463–1472. doi:10.1002/(SICI)1096-987X(199709)18:12<1463::AID-JCC4>3.0.CO;2-H

28. Miyamoto S, Kollman PA. SETTLE: an analytical version of the SHAKE and RATTLE algorithm for rigid water models. J Comput Chem. 1992;13: 952–962. Available: http://onlinelibrary.wiley.com/doi/10.1002/jcc.540130805/abstract

29. Bussi G, Donadio D, Parrinello M. Canonical sampling through velocity rescaling. J Chem Phys. Computational Science, Department of Chemistry and Applied Biosciences, ETH Zürich, USI Campus, Via Giuseppe Buffi 13, CH-6900 Lugano, Switzerland. gbussi@ethz.ch: American Institute of Physics; 2007;126: 14101. Available: http://eutils.ncbi.nlm.nih.gov/entrez/eutils/elink.fcgi?dbfrom=pubmed&id=17212484&retmode=ref&cmd=prlinks

30. Darden T, York D, Pedersen L. Particle mesh Ewald: An N log(N) method for Ewald sums in large systems. J Chem Phys. 1993;98: 10089. doi:10.1063/1.464397

31. Espinosa E, Molins E, Lecomte C. Hydrogen bond strengths revealed by topological analyses of experimentally observed electron densities. Chem Phys Lett. 1998;285: 170–173. Available: http://linkinghub.elsevier.com/retrieve/pii/S0009261498000360

32. Gallivan JP, Dougherty D a. Cation-pi interactions in structural biology. Proc Natl Acad Sci U S A. 1999;96: 9459–64. Available: http://www.pnas.org/cgi/doi/10.1073/pnas.96.17.9459

33. Kabsch W, Sander C. Dictionary of protein secondary structure: Pattern recognition of hydrogen-bonded and geometrical features. Biopolymers. 1983;22: 2577–2637. Available: http://doi.wiley.com/10.1002/bip.360221211

34. Aurora R, Rose GD. Helix capping. Protein Sci. 1998;7: 21–38. Available: http://www.pubmedcentral.nih.gov/articlerender.fcgi?artid=2143812&tool=pmcentrez&rendertype=abstract

35. Westfield GH, Rasmussen SGF, Su M, Dutta S, Devree BT, Chung KY, et al. Structural flexibility of the G alpha s alpha-helical domain in the beta2-adrenoceptor Gs complex. Proc Natl Acad Sci U S A. Life Sciences Institute and Department of

Biological Chemistry, University of Michigan Medical School, Ann Arbor, MI 48109, USA.; 2011;108: 16086–16091. Available: http://eutils.ncbi.nlm.nih.gov/entrez/eutils/elink.fcgi?dbfrom=pubmed&id=21914848&retmode=ref&cmd=prlinks

36. McDonald IK, Thornton JM. Satisfying hydrogen bonding potential in proteins. J Mol Biol. Department of Biochemistry and Molecular Biology, University College London, U.K.; 1994;238: 777–793. Available: http://www.sciencedirect.com/science?_ob=ArticleURL&_udi=B6WK7-45NSJV0-CW&_user=1853498&_coverDate=05/19/1994&_rdoc=1&_fmt=high&_orig=search&_sort=d&_docanchor=&view=c&_acct=C000055104&_version=1&_urlVersion=0&_userid=1853498&md5=7db20abaf65010262de88678422adecd

37. Wallace AC, Laskowski RA, Thornton JM. LIGPLOT: a program to generate schematic diagrams of protein-ligand interactions. Protein Eng. Department of Biochemistry and Molecular Biology, University College, London, UK.; 1995;8: 127–134. Available: http://eutils.ncbi.nlm.nih.gov/entrez/eutils/elink.fcgi?dbfrom=pubmed&id=7630882&retmode=ref&cmd=prlinks
